# Tbr1 Misexpression Alters Neuronal Development in the Cerebral Cortex

**DOI:** 10.1007/s12035-022-02936-x

**Published:** 2022-07-04

**Authors:** Inmaculada Crespo, Jaime Pignatelli, Veena Kinare, Héctor R. Méndez-Gómez, Miriam Esgleas, María José Román, Josep M. Canals, Shubha Tole, Carlos Vicario

**Affiliations:** 1 Instituto Cajal-Consejo Superior de Investigaciones Científicas (CSIC), Avenida Doctor Arce 37, 28002 Madrid, Spain; 2 CIBERNED-Instituto de Salud Carlos III (ISCIII), Madrid, Spain; 3 CES Cardenal Cisneros, Madrid, Spain; 4 Department of Life Sciences, Sophia College for Women, Mumbai, 400026 India; 5 Laboratory of Stem Cells and Regenerative Medicine, Department of Biomedical Sciences, Creatio, Production and Validation Center of Advanced Therapies, Faculty of Medicine and Health Sciences, Institute of Neurosciences, University of Barcelona, Barcelona, Spain; 6 August Pi I Sunyer Biomedical Research Institute (IDIBAPS), Barcelona, Spain; 7 Department of Biological Sciences, Tata Institute of Fundamental Research, Mumbai, 400005 India

**Keywords:** Tbr1, Cerebral cortex, Neuronal migration, Neuronal subtypes, Dendrite development, Callosal axons

## Abstract

Changes in the transcription factor (TF) expression are critical for brain development, and they may also underlie neurodevelopmental disorders. Indeed, T-box brain1 (Tbr1) is a TF crucial for the formation of neocortical layer VI, and mutations and microdeletions in that gene are associated with malformations in the human cerebral cortex, alterations that accompany autism spectrum disorder (ASD). Interestingly, Tbr1 upregulation has also been related to the occurrence of ASD-like symptoms, although limited studies have addressed the effect of increased Tbr1 levels during neocortical development. Here, we analysed the impact of Tbr1 misexpression in mouse neural progenitor cells (NPCs) at embryonic day 14.5 (E14.5), when they mainly generate neuronal layers II–IV. By E18.5, cells accumulated in the intermediate zone and in the deep cortical layers, whereas they became less abundant in the upper cortical layers. In accordance with this, the proportion of Sox5^+^ cells in layers V–VI increased, while that of Cux1^+^ cells in layers II–IV decreased. On postnatal day 7, fewer defects in migration were evident, although a higher proportion of Sox5^+^ cells were seen in the upper and deep layers. The abnormal neuronal migration could be partially due to the altered multipolar-bipolar neuron morphologies induced by Tbr1 misexpression, which also reduced dendrite growth and branching, and disrupted the *corpus callosum*. Our results indicate that Tbr1 misexpression in cortical NPCs delays or disrupts neuronal migration, neuronal specification, dendrite development and the formation of the callosal tract. Hence, genetic changes that provoke ectopic Tbr1 upregulation during development could provoke cortical brain malformations.

**Supplementary Information:**

The online version contains supplementary material available at 10.1007/s12035-022-02936-x.

## Introduction  

The cerebral cortex is a multi-layer structure that comprised glutamatergic projection neurons and GABAergic interneurons. Projection neurons of the cortical plate (CP) are generated from radial glial progenitors (RGPs) or from intermediate progenitors (IPs), which are commonly characterized by the expression of Pax6 and Tbr2, respectively, along with other molecular markers [1–4]. Once generated, immature neocortical projection neurons migrate from the ventricular and subventricular zones (VZ and SVZ), through the intermediate zone (IZ), which is where they first adopt a multipolar morphology with multiple neurite extensions. Subsequently, these neurons acquire a bipolar morphology, and they begin to migrate radially along the radial glia. Neurons reach the CP in an “inside-outside” order, first forming the deep cortical layers VI and V and then progressively seeding the upper cortical layers II–IV [4–7]. Corticofugal neurons remain largely confined to layers VI and V of the cortex, and their production decreases rapidly from E14.5 when the generation of upper layer neurons commences. Importantly, it is these neurons that extend intra- and interhemispheric projections, such as those that form the *corpus callosum* [8, 9].

All these processes are tightly controlled during development by transcription factors (TFs) like T-box brain 1 (Tbr1 or TES-56), which is expressed strongly in the developing dorsal telencephalon (neocortex, hippocampus and olfactory bulb (OB)). Tbr1 is expressed by newborn neocortical neurons, specifically Cajal-Retzius cells, subplate cells and layer VI glutamatergic neurons, as well as the cells in the developing hippocampus and OB [10–22]. Moreover, Tbr1 has also been detected in dividing NPCs, giving rise to OB mitral neurons [23, 24]

Tbr1 is necessary for the differentiation of the preplate and layer VI neurons, for axon pathfinding and for the correct acquisition of the regional and laminar identity of projection neurons [12, 25–29]. Tbr1 is part of a glutamatergic neurogenic TF cascade [1, 20], and several factors repress its expression and regulate its activity, including CTIP1 and Fezf2 in layer V neurons [27, 28, 30]. By contrast, Tbr1 can also be activated by other TFs like Satb2 [31]. Tbr1 binds to its coactivator CASK1 to induce the transcription of genes involved in ASD, including Reelin, Nmdar2b/Grin2b, Auts2, Foxp2 and Wnt7b [26, 32–35].

De novo *TBR1* mutations, microdeletions and variants causing loss-of-function of this TF are found recurrently in individuals with brain malformations that accompany ASD and intellectual disability [33, 36–51]. More recently, TBR1 upregulation was detected in the cerebral cortex of knock-in mice carrying the TBR1-K228E mutation [52], a mutation previously identified in ASD [37, 42]. Synaptic transmission is altered in these mice, which also present ASD-like deficits [52]. Moreover, TBR1 upregulation has also been demonstrated in neurons derived from induced pluripotent stem cells (iPSCs) obtained from a 13-year-old male with savant syndrome [53], a condition where prodigious talent can occur in conjunction with ASD [54]. Similarly, TBR1 upregulation has been reported in neurons derived from human embryonic stem cells (hESCs) carrying the CHD7 intronic variant identified in ASD individuals [55]. Accordingly, not only TBR1 loss-of-function but also abnormally increased TBR1 expression could potentially provoke brain malformations and functional deficits.

Accordingly, we studied here the impact of Tbr1 misexpression on NPC proliferation, neuronal migration, layer formation and neuronal morphology during embryonic and postnatal development of the mouse cerebral cortex. Our results show that ectopic Tbr1 overexpression produces an accumulation of neurons in the IZ and deep layers of the cerebral cortex in vivo, impairing and delaying cell migration to the upper layers, altering neuronal specification and disrupting the development of dendrites and the callosal axon tract. Our findings suggest that Tbr1 levels must be tightly regulated during cerebral cortex development to prevent the occurrence of neurodevelopmental disorders.

## Materials and Methods

### Cloning of a Plasmid Expressing Human Tbr1

Stable Tbr1 expression was achieved using a modified Moloney murine leukaemia virus-based retroviral vector [23] carrying a CAG promoter and a WPRE sequence. The resulting construct pCAG-Tbr1-IRES-EGFP-WPRE (hereafter pCAG-Tbr1-EGFP) was used in parallel with the control pCAG-IRES-EGFP-WPRE vector (hereafter pCAG-EGFP). The CAG promoter was removed from the pCAGGS plasmid (kindly provided by Dr. Jun-ichi-Miyazaki, Osaka University, Ibaraki, Osaka, Japan [56], with the SalI and XhoI restriction enzymes, and then cloned upstream of the human *Tbr1 ORF* or of *IRES* into the pRV-hTbr1-IRES-EGFP and pRV-IRES-EGFP plasmids, respectively [23], previously digested with XhoI. The WPRE region was cloned from a pLV-IRES-EGFP-WPRE vector (a kind gift of Dr. Pantelis Tsoulfas, The Miami Project to Cure Paralysis, Miami, FL, USA) by digesting it with SalI and EcoRI, and it was inserted into the plasmids pCAG-Tbr1-IRES-EGFP and pCAG-IRES-EGFP previously digested with SalI. After transforming bacteria (*Escherichia coli*) with the corresponding plasmids and extracting the DNA, a Tbr1 clone was selected free of mutations in the Tbr1 coding region. The two plasmids (pCAG-Tbr1-EGFP and pCAG-EGFP) were transfected into 293THEK cells to confirm the expression of both Tbr1 and EGFP, and these plasmids were used in the electroporation experiments.

### In Vivo Electroporation of the pCAG-Tbr1-EGFP and pCAG-EGFP Plasmids

For in utero electroporation, embryonic day (E) 14.5 pregnant CD1 mice were anaesthetized with isofluorane (Isoba vet, Schering-Plough/Merck) and placed on a thermal plate. The skin and the abdominal cavity were opened through a midline incision, and the uterine horns were exposed. The pCAG-Tbr1-EGFP and pCAG-EGFP plasmids (1 µg/µl) were mixed with 0.01% Fastgreen, and 2–5 µl of either solution was injected into the lateral ventricle of each embryo using a pulled glass micropipette. For electroporation, the head of the embryos was placed between 3-mm tweezer-type platinum disk electrodes (NEPAGENE, Japan), and five 33-V pulses of 50 ms were administered at 950-ms intervals using a NEPA21 electroporator (NEPA GENE, Chiba, Japan). The region electroporated was the ventricular zone in the dorso-lateral area of the cerebral cortex, which at E14.5 is enriched in NPCs committed to generate layer II–IV neurons [2, 4–7]. After injection, the uterus was repositioned, the cavity of the pregnant mother was sutured and the embryos were allowed to continue developing until E15.5, E18.5, or postnatal-day (P) 7. In all experiments, electroporation of pCAG-EGFP and pCAG-Tbr1-EGFP was performed in parallel, as was the analysis of the mice.

### IdU and CIdU Labelling

To study the effect of Tbr1 misexpression on NPC proliferation, pregnant mothers were injected intraperitoneally (i.p.) with IdU (57.65 mg/kg) 3 h after electroporation and with CIdU (42.75 mg/kg) 24 h after electroporation, and the embryos were then analysed at E18.5.

### Tissue Collection and Immunohistochemistry

Pregnant mice were anaesthetized by injection (i.p.) of ketamine/xylazine, and each embryo was then perfused transcardially with 0.9% NaCl and 4% paraformaldehyde (PFA), before removing their brain and post-fixing it in the same fixative solution for 2 days. E18.5 embryos were anaesthetized by placing them on ice prior to perfusion. Their brain was embedded in 3% agarose, and the coronal or sagittal vibratome Sects. (50 µm) were obtained and stored at 4 °C in phosphate-buffered saline (PBS) containing 0.02% sodium azide until use. P7 mice were anaesthetized by injection of ketamine/xylazine (i.p.) and perfused, and their brains were removed and processed as indicated above.

Sections containing the neocortex were immunostained with antibodies against the following: GFP (1:1000, rat: Nacalai Tesque Cat# 04,404–84, RRID:AB_10013361; 1:1000, rabbit, Molecular Probes Cat# A-6455, RRID:AB_221570), CDP/Cux1 (1:500, rabbit: Santa Cruz Cat# sc-13024, RRID:AB_2261231), CIdU (1:500, rat: Accuratechemicals Cat# OBT0030, RRID:AB_2313756: kindly shared with us by Dr J.L. Trejo, Instituto Cajal-CSIC, Madrid, Spain), cleaved Caspase-3 (1:300, rabbit: Cell signaling Cat# 9661, RRID:AB_2341188), CTIP2 (1:500, rat: Abcam Cat# ab18465, RRID:AB_2064130), IdU (1:500, mouse: BD Biosciences Cat# 347,580, RRID:AB_400326: kindly shared with us by Dr J.L. Trejo, Instituto Cajal-CSIC, Madrid, Spain), Ki67 (1:500, rabbit: Thermo Scientific Cat# RM-9106-S0, RRID:AB_2341197), neuronal nuclei antigen (NeuN, 1:50; mouse, Millipore Cat# MAB377, RRID:AB_2298772), Pax6 (1:300; rabbit, Covance Cat# PRB-278-P, RRID:AB_291612), RC2 (1:100, mouse: Developmental Studies Hybridoma Bank), Sox5 (1:500, rabbit: a kind gift from Dr. A.V. Morales, Instituto Cajal-CSIC, Madrid, Spain), Tbr1 (1:6000, rabbit: Abcam Cat# ab31940, RRID:AB_2200219) and Tbr2 (1:300, rabbit: Abcam Cat# ab23345, RRID:AB_778267).

The secondary antibodies used were Alexa-488-, Alexa-594- and Alexa-647-conjugated affinity-purified antibodies against rabbit, rat or mouse IgGs (1:1000: Invitrogen/Fisher and Molecular Probes) or biotin-conjugated anti-rabbit IgG (1:1200: Jackson ImmunoResearch), visualized with peroxidase-conjugated streptavidin (1:1200: Jackson ImmunoResearch) and tyramide (1:150: Cell Signaling). Finally, the sections were washed, exposed to Hoechst (1 µg/ml: Sigma) and mounted on glass microscope slides in Mowiol. The sections were co-stained with a GFP antibody to allow unambiguous visualization and examination of the EGFP-labelled cells. Controls were performed to confirm the specificity of the primary and secondary antibodies.

### Cell counting and Morphological and Statistical analysis

**Fig. 1:**
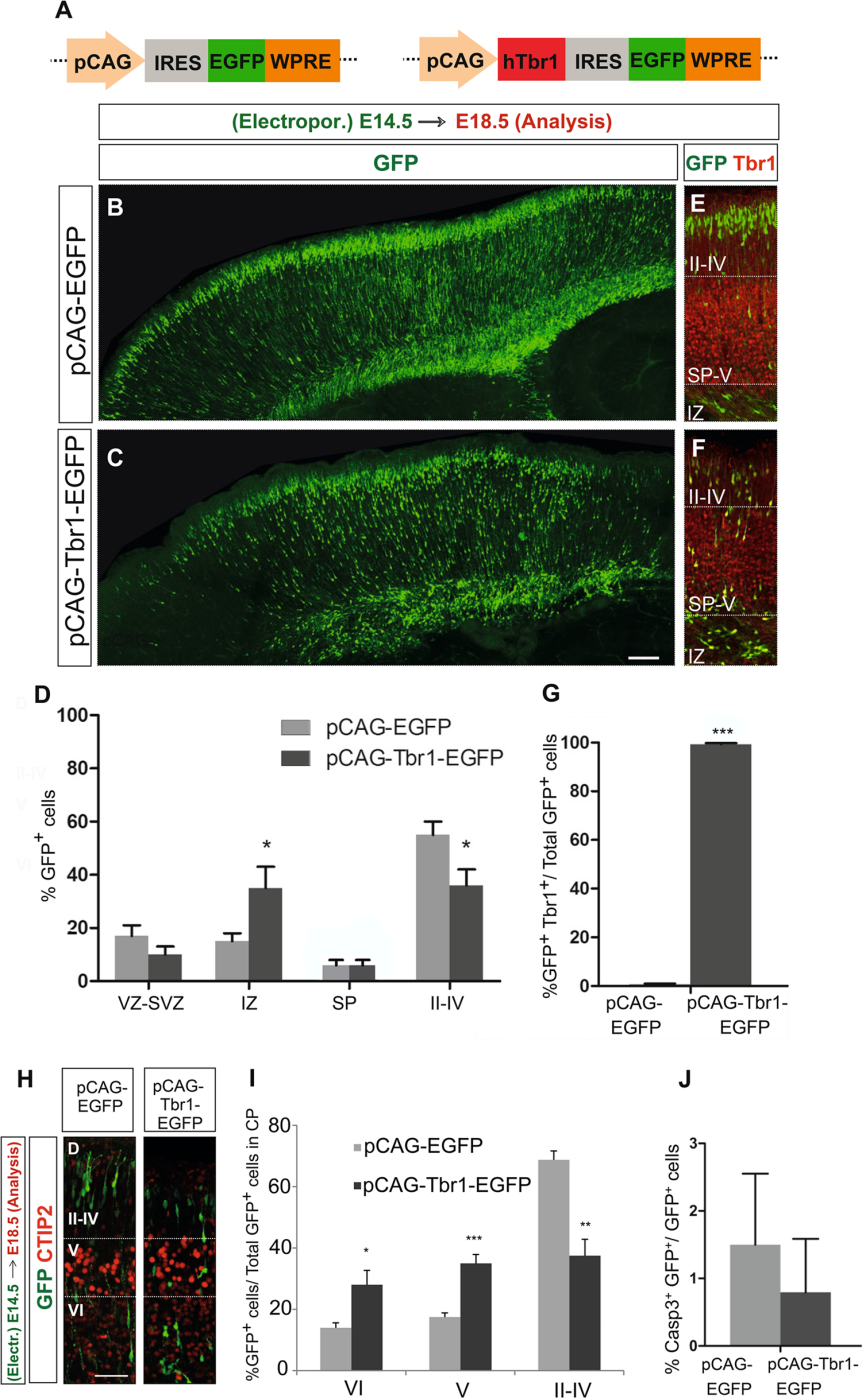
Tbr1 misexpression alters the distribution and positioning of migrating GFP^+^ cells in the cerebral cortex. **A** Scheme of the constructs used in the experiments. Mouse embryos were electroporated in utero at E14.5, their brain was collected at E18.5 and the vibratome sections were analysed by immunohistochemistry. A general view of the GFP^+^ cells in the sagittal sections from the cerebral cortex of embryos electroporated with the pCAG-EGFP (**B**) or pCAG-Tbr1-EGFP plasmid (**C**). **D** Quantification of the percentage of GFP^+^ cells in each zone and layer of the neocortex relative to the total number of GFP^+^ cells. **E**–**G** Dual GFP and Tbr1 immunohistochemistry of the coronal sections shows nearly 100% double-positive cells in the pCAG-Tbr1-EGFP electroporated brains compared to a very low number of these cells in the pCAG-EGFP animals (***P < 0.001; Student’s *t* test, *n* = 3 animals per condition). **H**, **I** Dual GFP and CTIP2 immunohistochemistry allows the percentage of GFP^+^ cells in layers VI, V and II–IV to be quantified accurately. Tbr1 misexpression produced a significant accumulation of GFP^+^ cells in the IZ and in layers VI and V, as well as a significant decrease of cells in layers II–IV. **J** The percentage of cleaved caspase 3.^+^ cells was very low, around 1% in both conditions (*n* = 3 animals per condition). The data in **D** and **I** are the mean ± S.E.M (*n* = 6–7 animals per condition): *P < 0.05, **P < 0.01, ***P < 0.001 (Student’s *t* test). Scale bar, 500 μm (**B**, **C**), 100 μm (**E**, **F**, **H**). VZ, ventricular zone; SVZ, subventricular zone; IZ, Intermediate zone; SP, subplate

Confocal images of individual *Z*-planes from five different rostro-caudal rectangular areas were taken with × 20, × 40 or × 63 objectives encompassing the whole ventro-dorsal cerebral cortex at a resolution of 1024 × 1024. Cells in the entire *Z*-stack from each area were counted manually to calculate the number of GFP^+^ cells, and co-localization of specific markers with GFP was analysed in each individual *Z*-plane using the ImageJ software (NIH, Bethesda, MD). The results in Figs. 1D and 6K are shown as the mean (± SEM) of the GFP^+^ cells found in a particular cortical zone or layer (counted as mentioned above) relative to the total number of GFP^+^ cells. Similarly, the mean (± SEM) number of multipolar, radial bipolar and non-radial bipolar GFP^+^ cells relative to the total number of GFP^+^ cells was recorded (Fig. 3). In all the experiments, 3–5 sections from 3–5 animals (*n*) per condition were examined for each immunostaining, except for Supplementary Fig. S1.

In addition, we counted the number of dendrites on GFP^+^ neurons to analyse the effect of Tbr1 overexpression on neuronal morphology in 63 × images from 15 neurons per condition. We also measured the length of the dendrites, and the length and thickness of the callosal axon fibres in the sections from E18.5 animals using the ImageJ software (NIH, Bethesda, MD, USA). Thresholds were established above the green background levels with the Image-Adjust-Threshold setting.

The length of dendrites and the area they occupied in sections from P7 animals were measured using the Imaris 8.4 software (Bitplane, Zurich, Switzerland). The *dendrite area* (*A*) is defined as the sum of the areas of all the dendrite segment edges. The area of an edge is defined as a surface area of a frustum (truncated cone), where *A* = Σ *A*(*i*), *i* = 0,…*n* − 1, *n* and *n* = number of edges of a segment. An unpaired two-tailed Student’s *t* test was used to compare the mean ± SEM values from the two experimental conditions. Welch’s correction was applied when the variances of both groups were significantly different, as indicated by the *F* test. Statistical significance was set at *P* < 0.05, and GraphPad Prism 5.0 was used for all the statistical analyses.

## Results

### Tbr1 Misexpression Alters the Distribution of GFP.^+^ Cells in the Embryonic Cerebral Cortex Without Affecting Neural Progenitor Cell Number

Tbr1 regulates neuronal differentiation, axonal pathfinding and laminar and regional identities in the cerebral cortex [12, 20, 29]. While de novo mutations and variants that cause TBR1 loss-of-function are found in individuals with ASD [36, 37, 42, 44] and TBR1 upregulation has also recently been implicated in this disorder, little is known about the mechanisms underlying such changes [52, 53, 55]. To study the effect of Tbr1 misexpression on NPCs largely predetermined to generate layer II–IV neurons, we performed in utero electroporation in E14.5 embryos and analysed the neocortex at E18.5 (Fig. 1A–J). Following electroporation of a bicistronic plasmid carrying the human Tbr1 cDNA (pCAG-Tbr1-EGFP), nearly 100% of GFP^+^ cells expressed Tbr1, whereas Tbr1-GFP^+^ cells were barely detected in embryos electroporated with the control pCAG-EGFP construct (Fig. 1E–G, *P* = 0.0001). The efficiency of electroporation of the two plasmids was similar, as evident in the number of EGFP^+^ cells detected (pCAG-EGFP, 144.41 ± 11.53; pCAG-Tbr1-EGFP, 179.27 ± 24.19). The GFP^+^ cells identified were distributed from the VZ and SVZ across to the upper cortical layers (Fig. 1B–D). However, Tbr1 overexpression produced a significant 2.3-fold increase (233%, *P* = 0.0317) in the percentage of cells located in the IZ in the GFP-immunostained sections, concomitant with a significant decrease (34.5%; *P* = 0.022) of the cells in layers II–IV (Fig. 1D).

As the number of GFP^+^ cells in layers VI and V could not be accurately counted, we determined the cell distribution in those layers (and in layers II–IV) using dual immunohistochemistry with antibodies against GFP and CTIP2, a marker of layer V neurons, which is also slightly expressed in layer VI neurons [30, 57] (Fig. 1H, I). Misexpression of Tbr1 at E14.5 significantly affected cell migration, with an accumulation of GFP^+^ cells in the deep layers at E18.5 (layer VI—14.0% ± 3.26 in the control animals, 28.0% ± 9.38 following Tbr1 overexpression, *P* = 0.030; layer V—17.5% ± 2.64 in control animals, 35.0% ± 5.71 following Tbr1 overexpression, *P* = 0.001) and fewer cells in the upper layers (layers II–IV—68.8% ± 5.79 in control animals, 37.5% ± 10.72 in Tbr1 animals, *P* = 0.002). By contrast, cell death was negligible and similar in both conditions (Fig. 1J). Together, these findings suggest that Tbr1 misexpression during embryonic development alters the migration and final position of the neurons derived from NPCs.

**Fig. 2:**
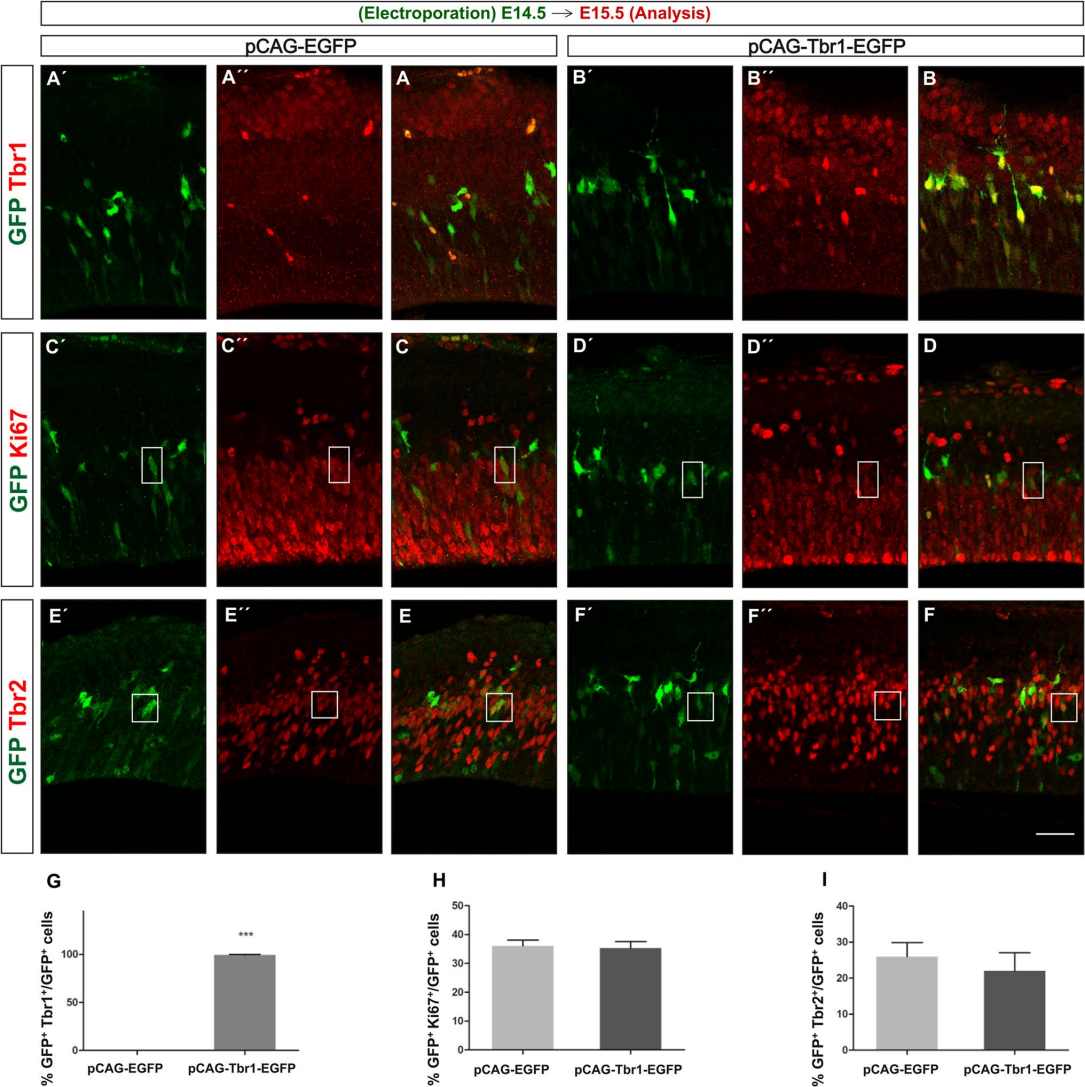
Neither cycling cells nor the intermediate progenitor (IP) population is affected by the Tbr1 misexpression. Mouse embryos were electroporated in utero at E14.5, their brain was collected at E15.5 and the coronal vibratome sections were analysed by dual immunohistochemistry. **A´**, **A´´**, **A**, **B´**, **B´´**, **B**, **G** The efficiency of Tbr1 overexpression was nearly 100% in GFP^+^ cells from pCAG-Tbr1-EGFP electroporated mice, whereas no GFP and Tbr1 double-labelled cells were detected in pCAG-EGFP electroporated mice. Tbr1 overexpression did not alter the percentage of GFP^+^Ki67^+^ cycling cells (**C´**, **C´´**, **C**, **D´**, **D´´**, **D**, **H**) nor that of GFP^+^Tbr2.^+^ IPs (**E´**, **E´´**, **E**, **F´**, **F´´**, **F**, **I**). Examples of the double labelled cells are shown in the rectangles and boxes, and the data are the mean ± S.E.M (*n* = 3–4 animals per condition). Scale bar, 20 μm

We then used a marker of the cell cycle, an antibody against Ki67, to check whether Tbr1 misexpression affects the number of actively cycling NPCs [58, 59]. No difference in the proportion of Ki67^+^ cells was observed when electroporation was performed at E14.5, and the embryos were analysed at E18.5 (data not shown). Indeed, when this assay was performed over a narrower time window, analysing the cerebral cortex of E14.5 electroporated embryos 24 h later (E15.5 embryos), GFP^+^ cells were detected in both conditions (Fig. 2). Moreover, in the embryos that received the pCAG-Tbr1-EGFP construct, nearly 100% of these GFP^+^ cells also expressed Tbr1 whereas GFP^+^-Tbr1^+^ cells were not found in the control condition (Fig. 2A´, B, G). Sections from these E15.5 embryos were then immunostained with antibodies against Ki67 and Tbr2, a TF widely expressed by IPs in the developing cerebral cortex [1, 60]. No significant differences in the percentages of GFP^+^ cells positive for Ki67 (*P* = 0.807: Fig. 2C´, D) or Tbr2 were evident between the two conditions (*P* = 0.553: Fig. 2E´, F). Thus, it appears that Tbr1 overexpression does not affect the number of cycling progenitors nor that of the IPs. To further explore whether proliferation is altered, pregnant mothers received IdU 3 h after electroporation and CIdU 24 h after electroporation. At E18.5, the percentages of double-labelled GFP^+^IdU^+^ and GFP^+^CIdU^+^ cells, as well as that of triple-labelled GFP^+^IdU^+^CIdU^+^ cells, were similar in both conditions, although only a small number of animals could be analysed (Supplementary Fig. S1); hence, Tbr1 misexpression appears not to alter the number of cells in S-phase nor that of cells staying in cell cycle from E14.5 to E18.5. In addition, Tbr1 misexpression did not significantly change the percentage of GFP^+^TuJ1^+^/GFP^+^ cells at E18.5 (data not shown) and almost 100% of GFP^+^ cells were NeuN^+^ at P7, both under control and Tbr1 misexpression conditions (Supplementary Fig. S2). These data suggest that neurogenesis was not affected by Tbr1 misexpression.

### Tbr1 Plays a Role Regulating Neuronal Polarization and Radial Cell Migration

**Fig. 3:**
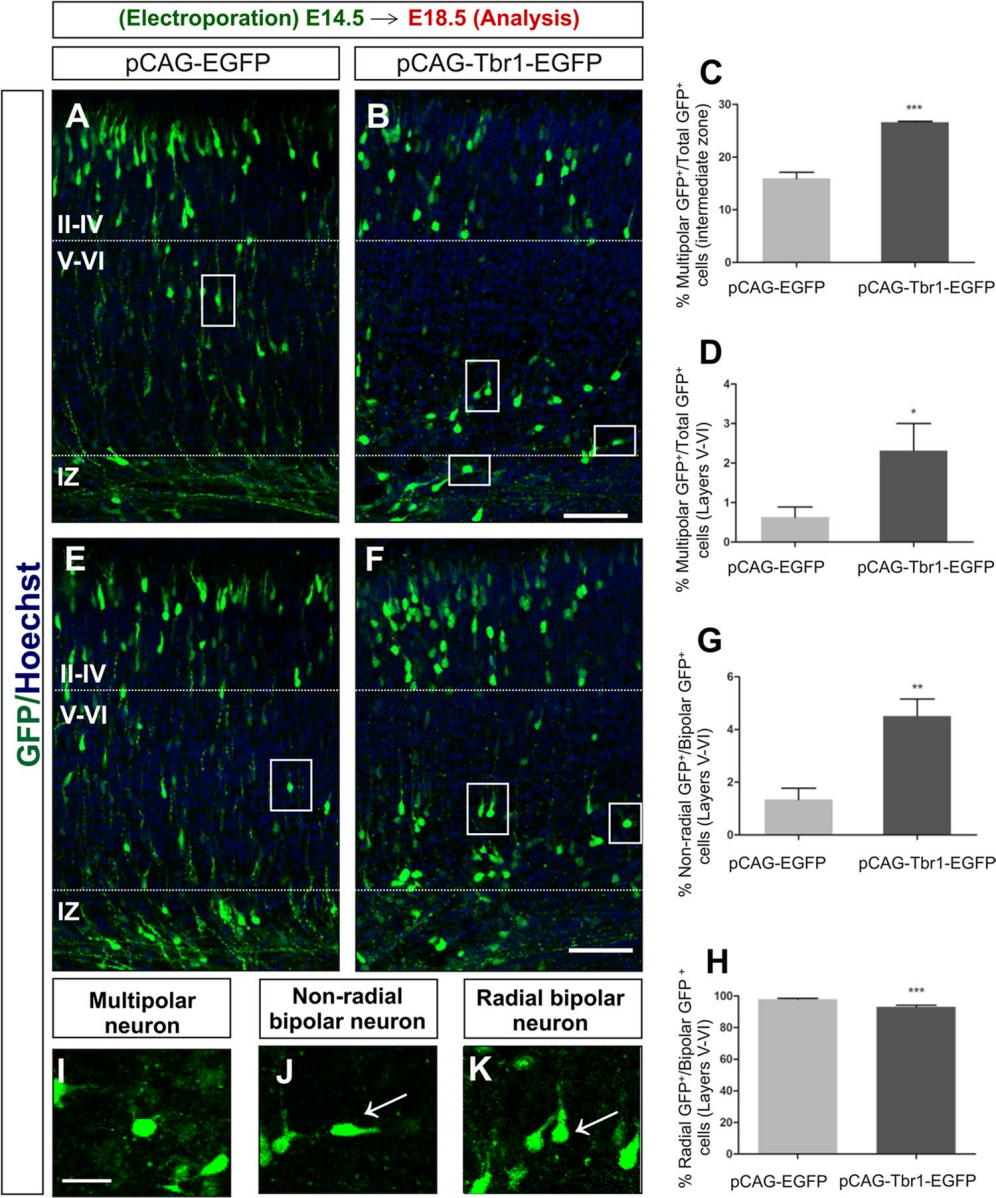
The effect of Tbr1 misexpression on the morphology and orientation of migrating neurons. The images show migrating neurons in the cortex of E18.5 animals that were electroporated with the pCAG-EGFP (**A**, **E**) or pCAG-Tbr1-EGFP plasmid (**B**, **F**). The high-magnification images show representative examples of multipolar (**I**), non-radial bipolar (**J**), and radial bipolar (**K**) GFP.^+^ immature neurons from the IZ and layers V–VI of embryos electroporated with the pCAG-Tbr1-EGFP plasmid (neurons are indicated by rectangles in **B** and **F**). The vibratome sections were immunostained with an anti-GFP antibody, and the quantification of the results is presented in the graphs. **C** Multipolar neurons in the IZ. **D** Multipolar neurons in layers V–VI. **G** Non-radial bipolar neurons in layers V–VI. **H** Radial bipolar neurons in layers V–VI. Note the significant increase in the percentages of multipolar and non-radial bipolar neurons after Tbr1 overexpression. The data are the mean ± S.E.M (*n* = 3 animals): *P < 0.05, **P < 0.01, ***P < 0.001 (Student’s *t* test). Scale bar, 20 μm

Since Tbr1 misexpression alters the distribution of neurons in the developing neocortex (Fig. 1), an ectopic increase in Tbr1 levels is likely to inhibit the migration of newly formed neurons. As such, we analysed neuronal polarization, given that immature projection neurons modify their morphology from multipolar to bipolar when crossing the IZ, becoming radially orientated [5–7, 61, 62]. Tbr1 misexpression significantly increased the proportion of multipolar neurons in the IZ (15.93% ± 1.17 in control, 26.60% ± 0.21 in Tbr1 overexpressing mice, *P* = 0.009) and in layers V–VI (0.62% ± 0.26 in control, 2.31% ± 0.68 in Tbr1 overexpressing mice, *P* = 0.032 (Fig. 3A–F, I). Furthermore, it increased the percentage of non-radial bipolar neurons in layers V–VI (1.34% ± 0.43 in control, 4.50% ± 0.65 in Tbr1 overexpressing mice, *P* = 0.002: Fig. 3G, J) and decreased the percentage of radial bipolar neurons (98.03% ± 0.56 in control, 93.17% ± 0.97 in Tbr1 overexpressing mice, *P* = 0.001: Fig. 3H, K). These data suggest that sustained Tbr1 expression in part disrupted neuronal migration by altering the correct transition from a multipolar to a radial bipolar state, which could slow their migration along radial glia. However, Tbr1 misexpression did not appear to change the radial glia scaffold (Supplementary Fig. S3) when it was analysed by immunostaining with an RC2 antibody that is a marker of radial glial [63].

### Tbr1 Misexpression Alters Neuron Subtype Specification in the Neocortex

**Fig. 4:**
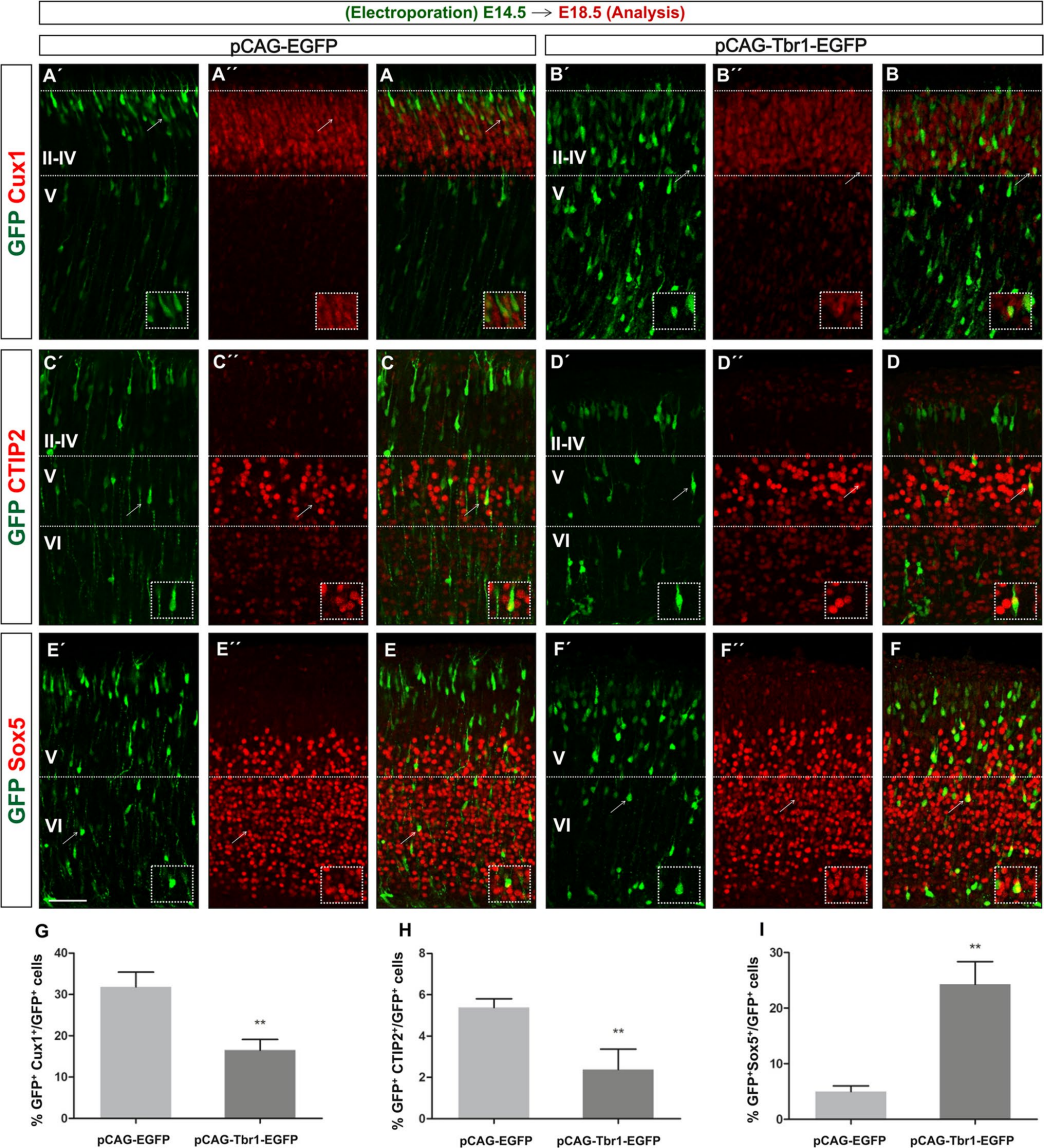
Tbr1 misexpression alters the neuronal specification and lamination. The coronal sections from E18.5 embryos electroporated with the pCAG-EGFP or pCAG-Tbr1-EGFP plasmid were dual immunostained with specific antibodies against GFP and Cux1 (**A´**, **A´´**, **A**, **B´**, **B´´**, **B**), CTIP2 (**C´**, **C´´**, **C**, **D´**, **D´´**, **D**), or Sox5 (**E´**, **E´´**, **E**, **F´**, **F´´**, **F**). The cells marked by arrows are shown at a higher magnification in the insets. Tbr1 overexpression significantly reduced the percentage of Cux1^+^ cells and CTIP^+^ cells in layers II–IV and V, respectively, while it increased the percentage of Sox5.^+^ cells in layers VI–V (**G**–**I**). The data are the mean ± S.E.M (*n* = 3–5 animals): *P < 0.05, **P < 0.01 (Student’s *t* test). Scale bar, 100 μm (**A**, **B**, **D**, **E**, **G**, **H**), 20 μm (insets)

In addition to producing defects in cell migration, Tbr1 misexpression might also affect the molecular identity of relevant neuronal subtypes. To assess this, we analysed the embryos electroporated at E14.5 and then immunostained at the E18.5 sections with antibodies against the following: Cux1, a marker of layer II–IV neurons [64]; CTIP2, a marker of layer V neurons [30, 57]; and Sox5, a marker of layer VI–V neurons [65] (Fig. 4A´–F´, A´´–F´´, A–F). Of the three neuronal subtypes studied here, Cux1^+^ neurons were those generated most abundantly between days E14.5 and E18.5 in the embryos electroporated with the control pCAG-EGFP construct (31.86% as opposed to 5.38% CTIP2^+^ cells and 4.99% Sox5^+^ cells (Fig. 4G–I). However, Tbr1 misexpression significantly decreased the proportion of Cux1^+^-GFP^+^ cells in layers II–IV (1.7-fold or 41.4%, *P* = 0.006: Fig. 4A´, B, G) and that of CTIP2^+^-GFP^+^ cells in layer V (2.4-fold or 58.0%, *P* = 0.031: Fig. 4C´, D, H), resulting in 18.74% Cux1^+^ and 2.24% CTIP2^+^ cells. By contrast, Tbr1 misexpression increased the percentage of Sox5^+^-GFP^+^ cells in layers V–VI (5.2-fold or 520%, *P* = 0.010: Fig. 4E´, F, I), resulting in 25.95% Sox5^+^ cells. The overall proportion of GFP^+^ cells expressing these three markers was 42.23% in the controls and 46.93% following Tbr1 overexpression. Hence, an increase in Tbr1 expression at these embryonic stages alters the neuronal subtype specification and cerebral cortex lamination.

### Dendrite and Axon Growth Is Also Affected by Tbr1 Misexpression

**Fig. 5:**
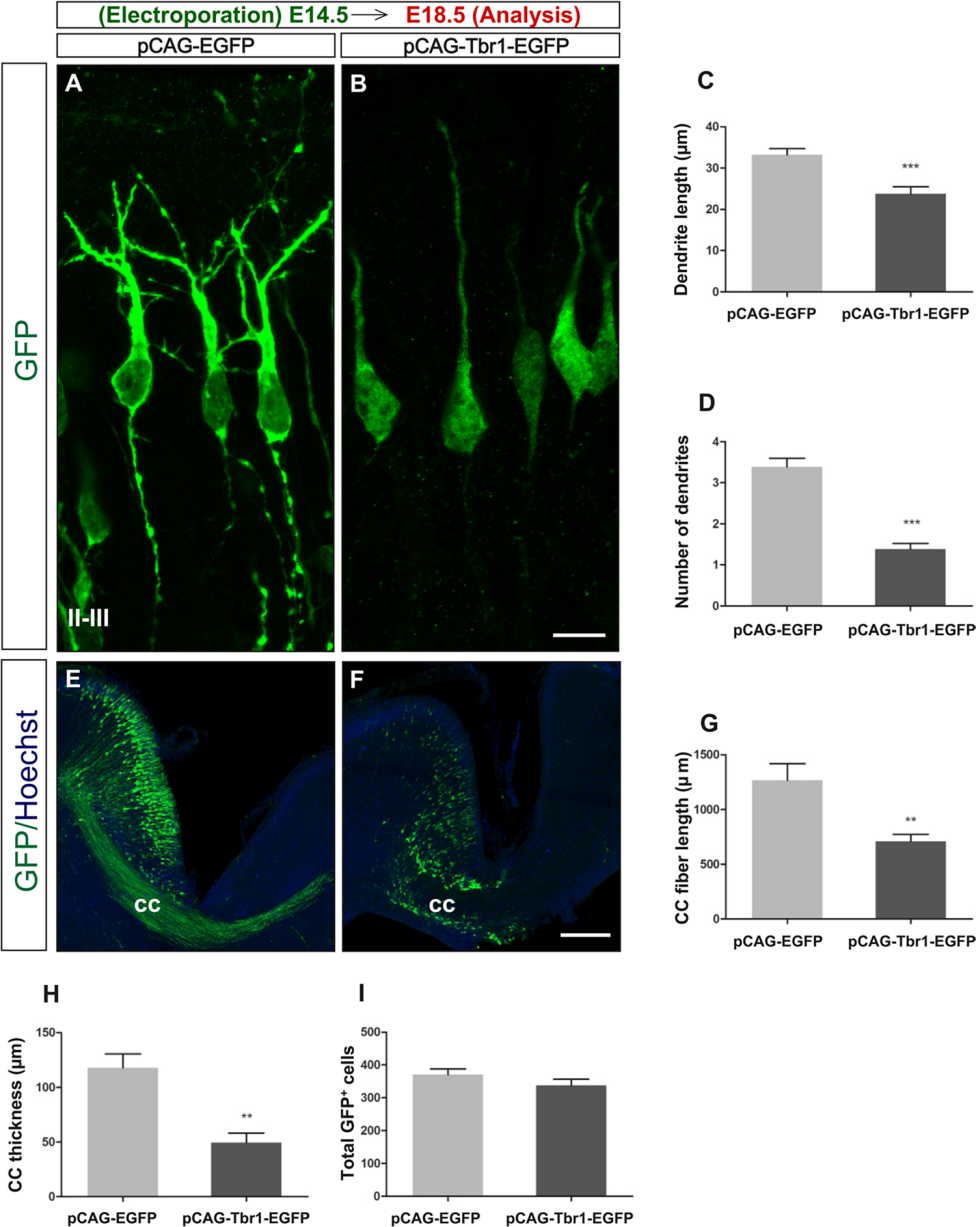
Tbr1 misexpression impairs dendrite and callosal axon development. The coronal vibratome sections from electroporated embryos were immunostained at E18.5 with a GFP antibody, and Hoechst was used to label the nuclei. Note that the complexity of neurons located in layers II–III (**A**, **B**) was markedly reduced in the embryos electroporated with pCAG-Tbr1-GFP, as indicated by the smaller dendrite length (*P* < 0.001; **C**) and the number of dendrites per neuron (*P* < 0.001; **D**); Student’s *t* test, *n* = 15 neurons per condition). **E**, **F** The images show axons forming the corpus callosum (CC) that cross to the contralateral hemisphere in the pCAG-EGFP electroporated animals, whereas this was markedly impaired following Tbr1 misexpression. The length and thickness of callosal fibres decreased significantly in the pCAG-Tbr1-EGFP electroporated animals (*P* < 0.01; Student’s *t* test; **G**, **H**). The total number of GFP.^+^ cells in the sections did not change (**I**). The data are the mean ± S.E.M (*n* = 3 animals per condition). Scale bar, 100 μm

We next studied whether ectopic Tbr1 overexpression affects dendrite and axon development in the neocortex. As such, we electroporated embryos at E14.5 and analysed the dendrite length and number at E18.5, as well as the callosal axon tract. We first observed that Tbr1 misexpression significantly decreased the length and the number of dendrites on layer II–III neurons (28.4%, *P* = 0.001 and 59.2%, *P* = 0.001, respectively: Fig. 5A–D). When the *corpus callosum* was analysed, the Tbr1 overexpression significantly reduced the callosal axon fibre length (44.07%, *P* = 0.008: Fig. 5E–G) and thickness (58.06%, *P* = 0.003: Fig. 5H), even though the total number of GFP electroporated cells was similar in both groups (*P* = 0.248; Fig. 5I). Thus, sustained Tbr1 misexpression impairs dendritogenesis, as well as callosal axon growth and guidance.

### Effect of Tbr1 Misexpression in Postnatal Neocortical Development

**Fig. 6:**
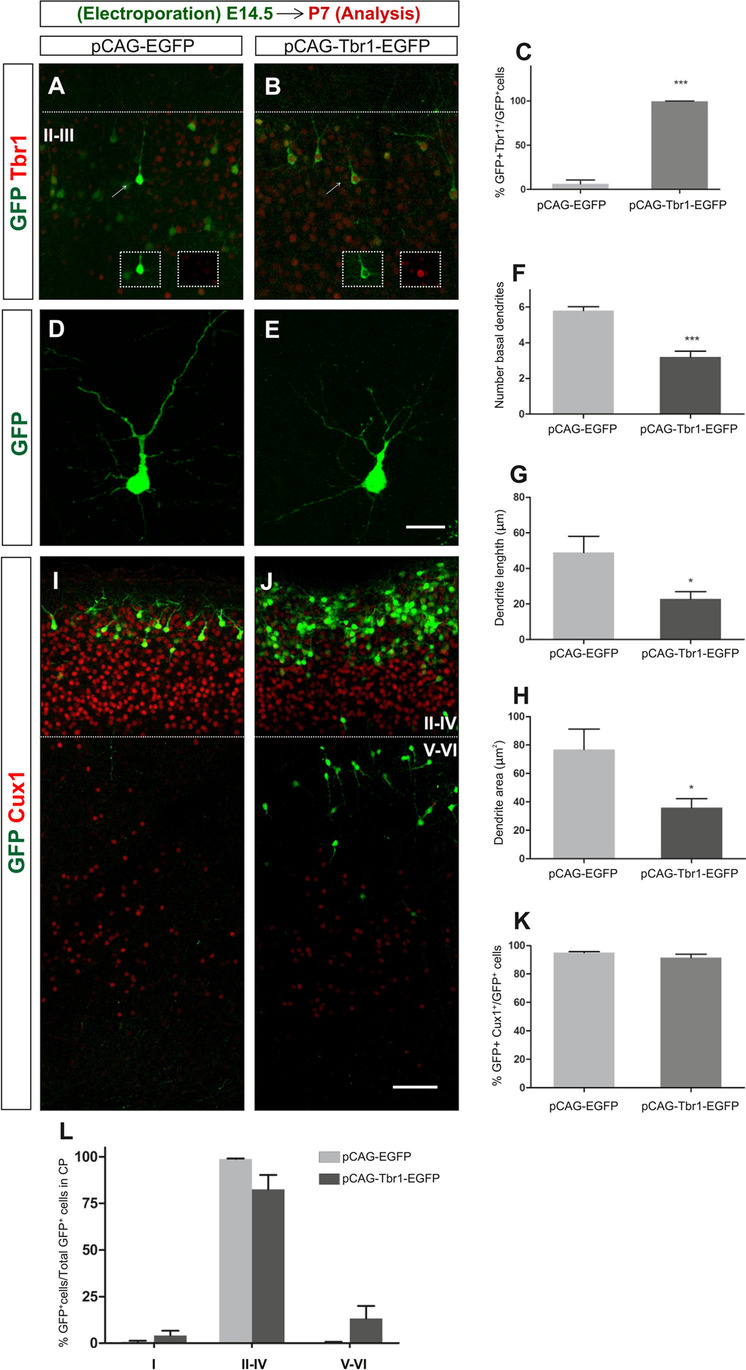
The altered dendrite morphology produced by Tbr1 misexpression is maintained in postnatal animals. Mouse embryos were electroporated in utero at E14.5, their brain was collected at P7 and the vibratome sections were immunostained. **A**–**C** Dual GFP and Tbr1 immunohistochemistry revealed nearly 100% of double-positive cells following pCAG-Tbr1-EGFP electroporation compared to the small number of these cells in the pCAG-EGFP electroporated animals (***P < 0.001; Student’s *t* test; *n* = 3 animals per condition). **D**–**H** Tbr1 overexpression significantly reduced the complexity of layer II–IV neurons in terms of the number of basal dendrites (*P* < 0.001; **F**), dendrite length (*P* < 0.05; **G**) and the area occupied by the dendrites (*P* < 0.05; **H**). The data in **F**–**H** are the mean ± S.E.M (*n* = 15 neurons per condition, Student’s *t* test). Scale bar, 100 μm. **I**–**K** To visualize the distribution of cells in the cortical layers, the P7 sections were immunostained with antibodies against GFP and Cux1 (**I**, **J**). **K** As seen in the graph, the percentages of GFP^+^Cux1^+^ cells were similar in control and Tbr1 overexpressing animals. **L** Almost 100% (98.8%) of the GFP^+^ cells were found in layers II–IV in the control animals whereas in the pCAG-Tbr1-EGFP electroporated animals, 82.5% and 13.3% of the GFP.^+^ cells were located in layers II–IV and V–VI, respectively. However, these differences did not achieve statistical significance. Scale bar, 200 μm

We next asked if the phenotypic alterations observed in Tbr1 electroporated embryos were maintained in postnatal animals, analysing P7 animals that were electroporated at E14.5. While at the developmental stages studied previously (E15.5 and E18.5) the proportion of GFP^+^-Tbr1^+^ cells relative to the GFP^+^ cells was close to 100% in P7 pCAG-Tbr1-EGFP electroporated mice, very few double-labelled cells were detected in the control (pCAG-EGFP) animals (*P* = 0.0001: Fig. 6A–C). It is important to note that at P7 Tbr1 expression it is not restricted to layer VI and layer V neurons, but it is also mildly expressed in the upper-layer neurons (Supplementary Fig. S4). We studied the impact of sustained Tbr1 expression on dendrites and found that it produced a significant reduction in the number of basal dendrites (including dendrite branches) per neuron in layers II–III (44.8%, *P* = 0.0001: Fig. 6D–F), as well as a reduction in dendrite length (53.3%, *P* = 0.015: Fig. 6G) and in the area occupied by the dendrites (53.22%, *P* = 0.015: Fig. 6H). These results indicate that Tbr1 misexpression alters the dendrite growth and number and that this effect persists in postnatal animals.

**Fig. 7:**
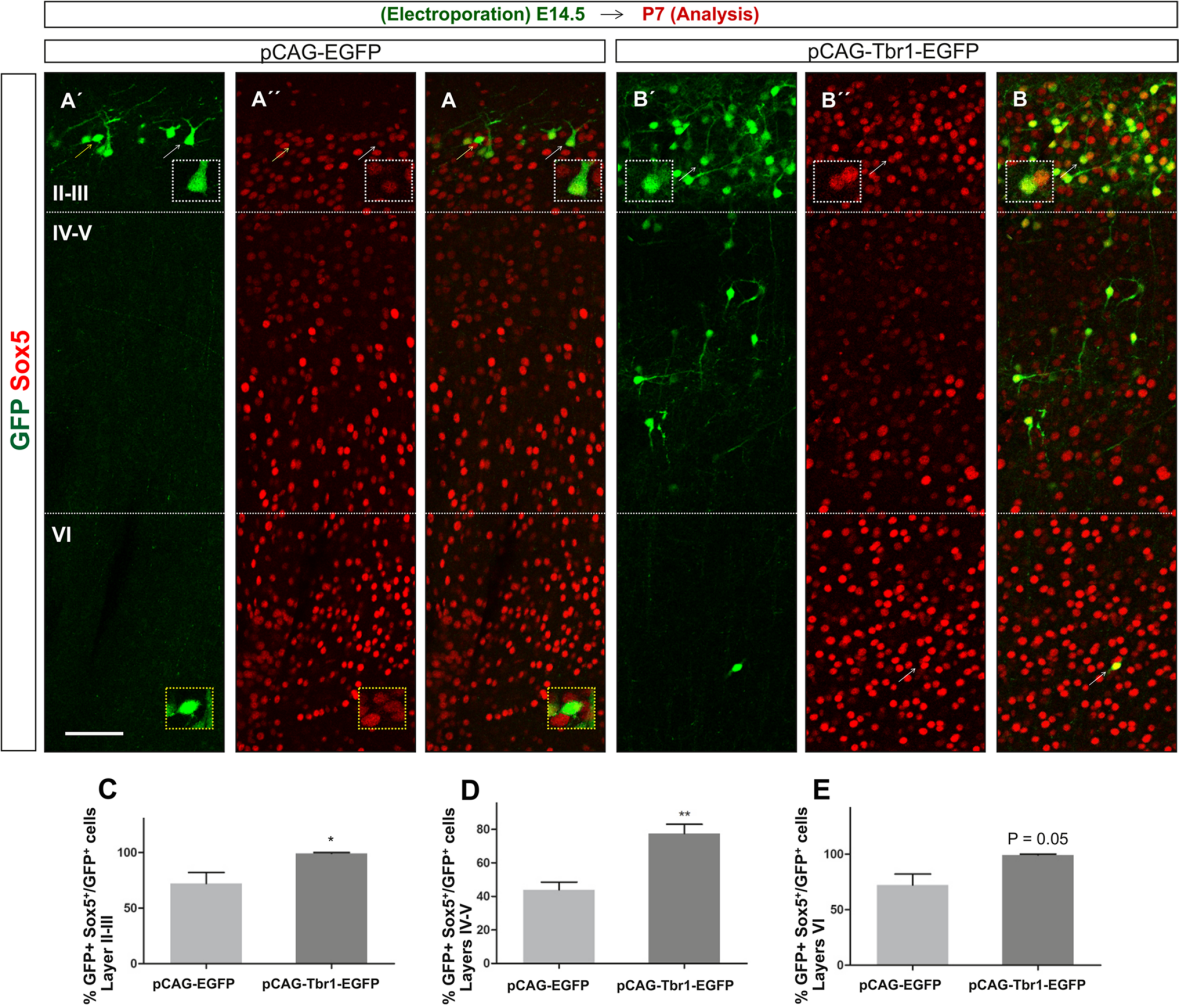
Tbr1 misexpression increases the proportion of Sox5 cells in the upper and deep layers of the neocortex. The coronal sections from P7 animals electroporated with the pCAG-EGFP or pCAG-Tbr1-EGFP plasmid were dual immunostained with specific antibodies against GFP and Sox5 (**A´**, **A´´**, **A**, **B´**, **B´´**, **B**). Examples of GFP^+^Sox5^+^ double-labelled cells are indicated with white arrows (**A´**, **A´´**, **A**, **B´**, **B´´**, **B** and top insets) whereas an example of GFP^+^Sox5^−^ cell is indicated with yellow arrows (**A´**, **A´´**, **A** and lower insets). As there were very few GFP^+^ cells from pCAG-EGFP electroporated mice in layer VI, these are not shown (see Fig. 6K). The cells marked by arrows in layers II–III are shown at a higher magnification in the insets. The pictures were composed of several images stitched together using the ImageJ software. Tbr1 overexpression increased the percentage of Sox5.^+^ cells in layers II–III (**C**, 70%, *P < 0.05 Student’s *t* test), layers IV–V (**D**, 77%, **P < 0.05 Student’s *t* test), and layer VI (**E**, 37%, *P* = 0.05). The data are the mean ± S.E.M (*n* = 3 animals per condition). Scale bar (shown in **A´**), 50 μm, 34.8 μm (insets)

To determine the distribution of GFP^+^ cells in the different layers of the cortex, the P7 sections were stained with antibodies to Cux1 in order to clearly distinguish GFP^+^ cells in layer I, from the upper (II–IV) and deep layers (V–VI: Fig. 6I, J). By P7, the vast majority of GFP^+^ cells were located in upper layers II–IV, and the labelled neurons were virtually absent from layers V–VI in the control mice. However, following Tbr1 overexpression 78% and 10% of GFP^+^ cells were detected in layers II–IV and layers V–VI, respectively, although these changes were not statistically significant (Fig. 6L). The percentages of GFP^+^Cux1^+^ cells were similar in both conditions (94.97% ± 0.7219 and 91.43% ± 2.554 in control and Tbr1 overexpressing animals, respectively) (Fig. 6K). By contrast, when cells that were immunostained with GFP and Sox5 antibodies were counted in sections from P7 mice, there were significantly higher proportions of GFP^+^Sox5^+^ cells in layers II–III (70%, *P* = 0.013, Fig. 7A–C) and in layers IV–V (76.6%, *P* = 0.0095) and a higher proportion of double-positive cells in layer VI (37%, *P* = 0.050, Fig. 7A, B, E) following Tbr1 misexpression. All these findings suggest that the defects in migration provoked by Tbr1 misexpression were not evident in P7 animals, although the alterations to the specification of Sox5 expressing neurons persisted. In addition, orientation defects in the targeted neurons that overexpress Tbr1 in lower layers can be observed in Figs. 6J and 7B´, B.

## Discussion

Loss-of-function studies have shown that Tbr1 is critical for the differentiation of Cajal-Retzius, subplate and layer VI neurons and for both neuron and laminar specification, as well as axon pathfinding [12, 25–31]. However, there is little evidence of the effects of ectopic Tbr1 overexpression, a fact that could be relevant to understand the aetiopathology of some ASD cases [52]. Here, we tested the impact of Tbr1 misexpression on NPCs and found defects in the development of glutamatergic projection neurons in the neocortex.

From previous studies [2, 6, 8] and following the electroporation of our control pCAG-EGFP plasmid, at E14.5, the vast majority of layer V (CTIP^+^) and layer VI (Tbr1^+^ and Sox5^+^) neurons have already been born, whereas layer II–IV neurons are still being generated. Thus, here, Tbr1 was misexpressed in NPCs that are mostly destined to produce layer II–IV projection neurons, which disturbed the correct pattern of neuronal migration, neuron subtype specification and dendrite development and that of the callosal axon tract. By contrast, cycling cells, the number of Tbr2^+^ progenitors and cell death were not significantly affected by Tbr1 misexpression in this manner. Tbr1 overexpressing cells accumulate in the IZ, and in layers VI and V, such that fewer neurons reach layers II–IV at E18.5. This defect in neuron migration to superficial layers under our conditions is somewhat similar to that reported for parvalbumin interneurons in a mutant mouse expressing higher levels of Tbr1 in the brain, although this effect could be secondary to changes that also affect projection neurons [52, 53, 55].

Our findings indicate significant increases in the proportion of immature multipolar neurons in both the IZ and layers V–VI, and that of non-radial bipolar neurons in layers V–VI, while the proportion of radial bipolar neurons decreases. These changes potentially explain the delay in neuronal migration caused by Tbr1 misexpression, best illustrated by the reduction of Cux1^+^ neurons in layers II–IV at E18.5 that it is not observed at P7. In fact, the transition from a multipolar to radial morphology of migrating neocortical neurons is critical for their migration along radial glial fibres in order to reach the cortical plate [5, 6, 61]. Although not studied here, it is tempting to speculate that Tbr1 misexpression could alter its association with CASK, which fulfils an important role during neuronal migration and morphological differentiation, partially due to the activation of reelin transcription by Tbr1 [12, 33, 34, 42, 66, 67]. Furthermore, our results show that the morphological alterations caused by Tbr1 misexpression not only affect migrating neurons but also they are observed in dendrites of neurons reaching layers II–III, as well as in callosal axons. These latter results suggest that Tbr1 upregulation could affect neuronal morphology, possibly by acting on genes that regulate cytoskeletal dynamics, neurite outgrowth and fasciculation [32, 35, 68]. The smaller number of neurons reaching layers II–IV might also explain the reduced thickness and length of the callosal axon tract in E18.5 brains following Tbr1 misexpression. The disturbance of migration at E18.5 is not significant at P7, suggesting a dampening of the effect of Tbr1, although dendrite morphology is still affected at this stage P7 such that some effects of Tbr1 misexpression appear to persist. Thus, the alterations to dendrites could have a negative effect on the connectivity of mature neurons, consequently affecting cortical networks.

The changes in neuronal lamination triggered by Tbr1 misexpression coincide with a decrease in the proportion of cells expressing molecular markers typical of layers II–IV (Cux1) and layer V (CTIP) and with an increase in the percentage of Sox5^+^ cells in layers VI–V at E18.5. Our results fit well with the phenotype found in the developing neocortex of Tbr1 null mutant mice in which the expression of layer II–IV markers increases, while that of layer VI markers diminishes [26–28]. Interestingly, our results are also consistent with the callosal defects reported in the Tbr1 null E18.5 mouse [12], and they support the concept that Tbr1 levels must be tightly regulated, both temporally and spatially, for the correct development of the cerebral cortex layers and the *corpus callosum*. The increase in the percentage of cells that accumulate in layer V when Tbr1 levels are sustained, concomitant with a decrease in the proportion of CTIP^+^ neurons, suggests that Tbr1 might partially repress CTIP, as reported previously [27, 30]. However, the increase in the number of Sox5^+^ neurons at P7 in the upper layers, when the defects in migration are less prominent, indicates that disruptions to the specification of these neurons are probably permanent. If so, the cortical upper layer will contain an imbalance in neuronal subtypes, as indicated by the similar numbers of Cux1^+^ cells and the larger number of Sox5^+^ cells at P7. Notably, it has been proposed that the dysregulation of specific genes in the upper-layer projection neurons, including SOX5, correlates with the clinical severity of ASD [77].

As mentioned above, mutations and microdeletions that cause TBR1 loss-of-function in humans are associated with cerebral cortex malformations that are accompanied by ASD and intellectual disability [33, 36, 44, 47, 48, 50, 51, 69]. This emphasizes the importance of TBR1 in human brain development and function. Indeed, *Tbr1*^+*/−*^ heterozygous mice display autism-like phenotypes, such as impaired social interactions, abnormal ultrasonic vocalization and defects in associative memory and cognitive flexibility [68, 70]. However, increased brain TBR1 levels have also recently been associated with altered synaptic transmission, ASD-like deficits and savant syndrome [52, 53, 55]. Importantly, neurons carrying the CHD7 intronic variant identified in ASD individuals displayed morphological defects that were rescued by downregulating TBR1, suggesting a causal effect of TBR1 upregulation in the defects reported [53]. Nonetheless, it will be necessary to perform additional studies to elucidate whether the deficits reported in these recent studies are a direct consequence of TBR1 upregulation.

Although our experimental strategy does not reproduce a genetic mutation that mimics the patient’s genome, our findings suggest that Tbr1 misexpression either delays or permanently disrupts neocortical neuronal migration, subtype specification, dendrite morphology and callosal axon formation. In fact, the abnormalities in neuron migration and in the development of callosal neuron processes in the upper-layer neurons detected here are compatible with the cortical-cortical connectivity failures observed in ASD individuals [33, 47, 71–75]. Moreover, our finding that neurons accumulate in the IZ is also compatible with the subcortical heterotopia characteristic of ASD brains [76].

In conclusion, together with previous results, our findings support the concept that Tbr1 levels must be finely regulated, both temporally and spatially, to prevent the occurrence of brain malformations that underlie ASD and intellectual disability.

## Supplementary Information

Below is the link to the electronic supplementary material.

Supplementary file1 (DOCX 296 KB)

Supplementary file2 (PDF 6494 KB)

## Data Availability

The data that support the findings of this study are available on reasonable request from the corresponding author.

## Declarations

### Research Involving Animals.

All animal care and handling were carried out in accordance with the European Union guidelines (directive 2010/63/EU) and Spanish legislation (Law 32/2007 and RD 53/2013). The protocols were approved by the Ethics Committees of the Consejo Superior de Investigaciones Científicas (CSIC, Madrid, Spain) and the Comunidad de Madrid (Spain). Some experiments related to the data shown in Fig. 1, as well as the Ki67 (at E18.5) and RC2 results, were performed at the Tata Institute of Fundamental Research following animal protocols approved by the Institution’s Animal Ethics Committee and according to the regulations devised by the Committee for the Purpose of Control and Supervision of Experiments on Animals. Animals were administered food and water ad libitum, and they were housed in strictly controlled environmental conditions: 12 h light/dark cycle, 22 °C temperature and 44% humidity. All efforts were made to ameliorate the animal’s suffering.
