# Supplementary material for: Tbr1 Misexpression Alters Neuronal Development in the Cerebral Cortex
Source: Mol Neurobiol. 2022 Jul 4;59(9):5750–65. doi: 10.1007/s12035-022-02936-x (PMC9395452; doi:10.1007/s12035-022-02936-x)
Supplement: Supplementary file 1 — Supplementary file1 (DOCX 296 KB) [file 12035_2022_2936_MOESM1_ESM.docx]

**Tbr1 misexpression alters neuronal development in the cerebral cortex**

Inmaculada Crespo^1,2,3^, Jaime Pignatelli^1,2^, Veena Kinare^4^, Héctor R. Méndez-Gómez^1,2^, Miriam Esgleas^2,5,6^, María José Román^1,2^, Josep M. Canals^2,5,6^, Shubha Tole^7^, Carlos Vicario^1,2, #^

^1^Instituto Cajal-Consejo Superior de Investigaciones Científicas (CSIC), Madrid, Spain; ^2^CIBERNED-Instituto de Salud Carlos III (ISCIII), Madrid, Spain; ^3^CES Cardenal Cisneros, Madrid, Spain; ^4^Department of Life Sciences, Sophia College for Women, Mumbai 400026, India; ^5^Laboratory of Stem Cells and Regenerative Medicine, Department of Biomedical Sciences, Creatio, Production and Validation Center of Advanced Therapies, Faculty of Medicine and Health Sciences, Institute of Neurosciences, University of Barcelona, Barcelona, Spain; ^6^August Pi i Sunyer Biomedical Research Institute (IDIBAPS), Barcelona, Spain; ^7^Department of Biological Sciences, Tata Institute of Fundamental Research, Mumbai 400005, India.

**^#^Corresponding author:** Carlos Vicario, Instituto Cajal-CSIC, Avenida Doctor Arce 37, E-28002 Madrid, Spain. Email: cvicario@cajal.csic.es. Phone: (+34) 91-585-4721; Fax: (+34) 91-585-4754

**Running Title:** Tbr1 misexpression alters cerebral cortex development

**Abstract**

Changes in transcription factor (TF) expression are critical for brain development and they may also underlie neurodevelopmental disorders. Indeed, T-box brain1 (Tbr1) is a TF crucial for the formation of neocortical layer VI, and mutations and microdeletions in that gene are associated with malformations in the human cerebral cortex, alterations that accompany autism spectrum disorder (ASD). Interestingly, Tbr1 upregulation has also been related to the occurrence of ASD-like symptoms, although limited studies have addressed the effect of increased Tbr1 levels during neocortical development. Here we analysed the impact of Tbr1 misexpression in mouse neural progenitor cells (NPCs) at embryonic day 14.5 (E14.5), when they mainly generate neuronal layers II-IV. By E18.5, cells accumulated in the intermediate zone and in the deep cortical layers, whereas they became less abundant in the upper cortical layers. In accordance with this, the proportion of Sox5^+^ cells in layers V-VI increased, while that of Cux1^+^ cells in layers II-IV decreased. On postnatal day 7, fewer defects in migration were evident, although a higher proportion of Sox5^+^ cells were seen in the upper and deep layers. The abnormal neuronal migration could be partially due to the altered multipolar-bipolar neuron morphologies induced by Tbr1 misexpression, which also reduced dendrite growth and branching, and disrupted the *corpus callosum*. Our results indicate that Tbr1 misexpression in cortical NPCs delays or disrupts neuronal migration, neuronal specification, dendrite development and the formation of the callosal tract. Hence, genetic changes that provoke ectopic Tbr1 upregulation during development could provoke cortical brain malformations.

**Key words:** Tbr1; cerebral cortex; neuronal migration; neuronal subtypes; dendrite development; callosal axons.

**Introduction**

The cerebral cortex is a multi-layer structure that is comprised of glutamatergic projection neurons and GABAergic interneurons. Projection neurons of the cortical plate (CP) are generated from radial glial progenitors (RGPs) or from intermediate progenitors (IPs), which are commonly characterized by the expression of Pax6 and Tbr2, respectively, along with other molecular markers [1-4]. Once generated, immature neocortical projection neurons migrate from the ventricular and subventricular zones (VZ and SVZ), through the intermediate zone (IZ), which is where they first adopt a multipolar morphology with multiple neurite extensions. Subsequently, these neurons acquire a bipolar morphology and they begin to migrate radially along radial glia. Neurons reach the CP in an “inside-outside” order, first forming the deep cortical layers VI and V, and then progressively seeding the upper cortical layers II-IV [4-7]. Corticofugal neurons remain largely confined to layers VI and V of the cortex, and their production decreases rapidly from E14.5 when the generation of upper layer neurons commences. Importantly, it is these neurons that extend intra- and interhemispheric projections, such as those that form the *corpus callosum* [8,9].

All these processes are tightly controlled during development by transcriptions factors (TFs) like T-box brain 1 (Tbr1 or TES-56), which is expressed strongly in the developing dorsal telencephalon (neocortex, hippocampus and olfactory bulb -OB). Tbr1 is expressed by new-born neocortical neurons, specifically Cajal-Retzius cells, subplate cells and layer VI glutamatergic neurons, as well as the cells in the developing hippocampus and OB [10-22]. Moreover, Tbr1 has also been detected in dividing NPCs, giving rise to OB mitral neurons [23,24]

Tbr1 is necessary for the differentiation of preplate and layer VI neurons, for axon pathfinding, and for the correct acquisition of the regional and laminar identity of projection neurons [12,25-29]. Tbr1 is part of a glutamatergic neurogenic TF cascade [1,20], and several factors repress its expression and regulate its activity, including CTIP1 and Fezf2 in layer V neurons [27,28,30]. By contrast, Tbr1 can also be activated by other TFs like Satb2 [31]. Tbr1 binds to its coactivator CASK1 to induce the transcription of genes involved in ASD, including Reelin, Nmdar2b/Grin2b, Auts2, Foxp2 and Wnt7b [26,32-35].

*De novo* *TBR1* mutations, microdeletions and variants causing loss-of-function of this TF are found recurrently in individuals with brain malformations that accompany ASD and intellectual disability [33,36-51]. More recently, TBR1 upregulation was detected in the cerebral cortex of knock-in mice carrying the TBR1-K228E mutation [52], a mutation previously identified in ASD [37,42]. Synaptic transmission is altered in these mice, which also present ASD-like deficits [52]. Moreover, TBR1 upregulation has also been demonstrated in neurons derived from iPSCs (induced Pluripotent Stem Cells) obtained from a 13-year-old male with savant syndrome [53], a condition where prodigious talent can occur in conjunction with ASD [54]. Similarly, TBR1 upregulation has been reported in neurons derived from human embryonic stem cells (hESCs) carrying the CHD7 intronic variant identified in ASD individuals [55]. Accordingly, not only TBR1 loss-of-function but also abnormally increased TBR1 expression could potentially provoke brain malformations and functional deficits.

Accordingly, we studied here the impact of Tbr1 misexpression on NPC proliferation, neuronal migration, layer formation and neuronal morphology during embryonic and postnatal development of the mouse cerebral cortex. Our results show that ectopic Tbr1 overexpression produces an accumulation of neurons in the IZ and deep layers of the cerebral cortex *in vivo*, impairing and delaying cell migration to the upper layers, altering neuronal specification, and disrupting the development of dendrites and the callosal axon tract. Our findings suggest that Tbr1 levels must be tightly regulated during cerebral cortex development to prevent the occurrence of neurodevelopmental disorders.

**Materials and Methods**

**Cloning of a plasmid expressing human Tbr1**

Stable Tbr1 expression was achieved using a modified Moloney murine leukaemia virus-based retroviral vector [23] carrying a CAG promoter and a WPRE sequence. The resulting construct pCAG-Tbr1-IRES-EGFP-WPRE (hereafter pCAG-Tbr1-EGFP) was used in parallel with the control pCAG-IRES-EGFP-WPRE vector (hereafter pCAG-EGFP). The CAG promoter was removed from the pCAGGS plasmid (kindly provided by Dr Jun-ichi-Miyazaki, Osaka University; Ibaraki, Osaka, Japan [56] with the SalI and XhoI restriction enzymes, and then cloned upstream of the human *Tbr1 ORF* or of *IRES* into the pRV-hTbr1-IRES-EGFP and pRV-IRES-EGFP plasmids, respectively [23] previously digested with XhoI. The WPRE region was cloned from a pLV-IRES-EGFP-WPRE vector (a kind gift of Dr. Pantelis Tsoulfas, The Miami Project to Cure Paralysis, Miami, Florida, USA) by digesting it with SalI and EcoRI, and it was inserted into the plasmids pCAG-Tbr1-IRES-EGFP and pCAG-IRES-EGFP previously digested with SalI. After transforming bacteria (*E. Coli*) with the corresponding plasmids and extracting the DNA, a Tbr1 clone was selected free of mutations in the Tbr1 coding region. The two plasmids (pCAG-Tbr1-EGFP and pCAG-EGFP) were transfected into 293THEK cells to confirm the expression of both Tbr1 and EGFP, and these plasmids were used in the electroporation experiments.

***In vivo* electroporation of the pCAG-Tbr1-EGFP and pCAG-EGFP plasmids**

For *in utero* electroporation, embryonic day (E) 14.5 pregnant CD1 mice were anesthetized with isofluorane (Isoba vet, Schering-Plough/Merck) and placed on a thermal plate. The skin and the abdominal cavity were opened through a midline incision and the uterine horns exposed. The pCAG-Tbr1-EGFP and pCAG-EGFP plasmids (1 µg/µl) were mixed with 0.01% Fastgreen, and 2-5 µl of either solution was injected into the lateral ventricle of each embryo using a pulled glass micropipette. For electroporation, the head of the embryos was placed between 3 mm tweezer-type platinum disk electrodes (NEPAGENE, Japan) and five 33V pulses of 50 milliseconds (ms) were administered at 950 ms intervals using a NEPA21 electroporator (NEPA GENE, Chiba, Japan). The region electroporated was the ventricular zone in the dorso-lateral area of the cerebral cortex, which at E14.5 is enriched in NPCs committed to generate layer II-IV neurons [2,4-7]. After injection, the uterus was repositioned, the cavity of the pregnant mother was sutured and the embryos were allowed to continue developing until E15.5, E18.5, or postnatal-day (P) 7. In all experiments, electroporation of pCAG-EGFP and pCAG-Tbr1-EGFP was performed in parallel, as was the analysis of the mice.

**IdU and CIdU labelling**

To study the effect of Tbr1 misexpression on NPC proliferation, pregnant mothers were injected intraperitoneally (i.p.) with IdU (57.65 mg/Kg) 3 hours after electroporation, and with CIdU (42.75 mg/Kg) 24 hours after electroporation, and the embryos were then analysed at E18.5.

**Tissue collection and immunohistochemistry**

Pregnant mice were anaesthetized by injection (i.p.) of ketamine/xylazine, and each embryo was then perfused transcardially with 0.9% NaCl and 4% paraformaldehyde (PFA), before removing their brain and post-fixing it in the same fixative solution for 2 days. E18.5 embryos were anesthetized by placing them on ice prior to perfusion. Their brain was embedded in 3% agarose and coronal or sagittal vibratome sections (50 µm) were obtained and stored at 4 ºC in phosphate buffered saline (PBS) containing 0.02% sodium azide until use. P7 mice were anaesthetized by injection of ketamine/xylazine (i.p.), perfused, and their brains were removed and processed as indicated above.

Sections containing the neocortex were immunostained with antibodies against: GFP (1:1000, rat: Nacalai Tesque Cat# 04404-84, RRID:AB_10013361; 1:1000, rabbit, Molecular Probes Cat# A-6455, RRID:AB_221570); CDP/Cux1 (1:500, rabbit: Santa Cruz Cat# sc-13024, RRID:AB_2261231); CIdU (1:500, rat: Accuratechemicals Cat# OBT0030, RRID:AB_2313756: kindly shared with us by Dr J.L. Trejo, Instituto Cajal-CSIC, Madrid, Spain); Cleaved Caspase-3 (1:300, rabbit: Cell signaling Cat# 9661, RRID:AB_2341188); CTIP2 (1:500, rat: Abcam Cat# ab18465, RRID:AB_2064130); IdU (1:500, mouse: BD Biosciences Cat# 347580, RRID:AB_400326: kindly shared with us by Dr J.L. Trejo, Instituto Cajal-CSIC, Madrid, Spain); Ki67 (1:500, rabbit: Thermo Scientific Cat# RM-9106-S0, RRID:AB_2341197); Neuronal nuclei antigen (NeuN , 1:50; mouse, Millipore Cat# MAB377, RRID:AB_2298772); Pax6 (1:300; rabbit, Covance Cat# PRB-278-P, RRID:AB_291612); RC2 (1:100, mouse: Developmental Studies Hybridoma Bank); Sox5 (1:500, rabbit: a kind gift from Dr A.V. Morales, Instituto Cajal-CSIC, Madrid, Spain); Tbr1 (1:6000, rabbit: Abcam Cat# ab31940, RRID:AB_2200219); and Tbr2 (1:300, rabbit: Abcam Cat# ab23345, RRID:AB_778267).

The secondary antibodies used were Alexa-488, Alexa-594 and Alexa-647 conjugated affinity-purified antibodies against rabbit, rat or mouse IgGs (1:1000: Invitrogen/Fisher and Molecular Probes), or biotin-conjugated anti-rabbit IgG (1:1200: Jackson ImmunoResearch), visualised with peroxidase-conjugated streptavidin (1:1200: Jackson ImmunoResearch) and tyramide (1:150: Cell Signaling). Finally, the sections were washed, exposed to Hoechst (1 µg/ml: Sigma) and mounted on glass microscope slides in Mowiol. The sections were co-stained with a GFP antibody to allow unambiguous visualization and examination of the EGFP-labelled cells. Controls were performed to confirm the specificity of the primary and secondary antibodies.

**Cell counting, morphological and statistical analysis**

Confocal images of individual z-planes from five different rostro-caudal rectangular areas were taken with 20x, 40x or 63x objectives encompassing the whole ventro-dorsal cerebral cortex at a resolution of 1024 x 1024. Cells in the entire Z-stack from each area were counted manually to calculate the number of GFP^+^ cells and co-localisation of specific markers with GFP was analysed in each individual Z-plane using ImageJ software (NIH, Bethesda; MD). Results in Fig. 1D and Fig. 6K are shown as the mean (±SEM) of the GFP^+^ cells found in a particular cortical zone or layer (counted as mentioned above) relative to the total number of GFP^+^ cells. Similarly, the mean (±SEM) number of multipolar, radial bipolar and non-radial bipolar GFP^+^ cells relative to the total number of GFP^+^ cells was recorded (Fig. 3). In all the experiments, 3-5 sections from 3-5 animals (“n”) per condition were examined for each immunostaining, except for Supplementary Fig. S1.

In addition, we counted the number of dendrites on GFP^+^ neurons to analyse the effect of Tbr1 overexpression on neuronal morphology in 63x images from 15 neurons per condition. We also measured the length of the dendrites, and the length and thickness of the callosal axon fibres in sections from E18.5 animals using ImageJ software (NIH, Bethesda, MD, USA). Thresholds were established above the green background levels with the Image-Adjust-Threshold setting.

The length of dendrites and the area they occupied in sections from P7 animals was measured using Imaris 8.4 software (Bitplane, Zurich, Switzerland). The *dendrite area (A)* is defined as the sum of the areas of all the dendrite segment edges. The area of an edge is defined as a surface area of a frustum (truncated cone), where A = Σ A(i), i = 0,...n - 1, n and n = number of edges of a segment. An unpaired two-tailed Student’s *t*-test was used to compare the mean ± SEM values from the two experimental conditions. Welch´s correction was applied when the variances of both groups were significantly different, as indicated by the F test. Statistical significance was set at *P*<0.05 and GraphPad Prism 5.0 was used for all the statistical analyses.

**Results**

**Tbr1 misexpression alters the distribution of GFP^+^ cells in the embryonic cerebral cortex without affecting neural progenitor cell number**

Tbr1 regulates neuronal differentiation, axonal pathfinding, and laminar and regional identities in the cerebral cortex [12,20,29]. While *de novo* mutations and variants that cause TBR1 loss-of-function are found in individuals with ASD [36,37,42,44] and TBR1 upregulation has also recently been implicated in this disorder, little is known about the mechanisms underlying such changes [52,53,55]. To study the effect of Tbr1 misexpression on NPCs largely predetermined to generate layer II-IV neurons, we performed *in utero* electroporation in E14.5 embryos and analysed the neocortex at E18.5 (Fig. 1A-J). Following electroporation of a bicistronic plasmid carrying the human Tbr1 cDNA (pCAG-Tbr1-EGFP), nearly 100% of GFP^+^ cells expressed Tbr1, whereas Tbr1-GFP^+^ cells were barely detected in embryos electroporated with the control pCAG-EGFP construct (Fig. 1E-G, *P*=0.0001). The efficiency of electroporation of the two plasmids was similar, as evident in the number of EGFP^+^ cells detected (pCAG-EGFP, 144.41 ± 11.53; pCAG-Tbr1-EGFP, 179.27 ± 24.19). The GFP^+^ cells identified were distributed from the VZ and SVZ across to the upper cortical layers (Fig. 1B-D). However, Tbr1 overexpression produced a significant 2.3-fold increase (233%, *P*= 0.0317) in the percentage of cells located in the IZ in GFP immunostained sections, concomitant with a significant decrease (34.5%; *P*= 0.022) of the cells in layers II-IV (Fig. 1D).

As the number of GFP^+^ cells in layers VI and V could not be accurately counted, we determined the cell distribution in those layers (and in layers II-IV) using dual immunohistochemistry with antibodies against GFP and CTIP2, a marker of layer V neurons, which is also slightly expressed in layer VI neurons [57,30] (Fig. 1H-I). Misexpression of Tbr1 at E14.5 significantly affected cell migration, with an accumulation of GFP^+^ cells in the deep layers at E18.5 (layer VI - 14.0% ± 3.26 in the control animals, 28.0% ± 9.38 following Tbr1 overexpression, *P*=0.030; and layer V - 17.5% ± 2.64 in control animals, 35.0% ± 5.71 following Tbr1 overexpression, *P*=0.001) and fewer cells in the upper layers (layers II-IV - 68.8% ± 5.79 in control animals, 37.5% ± 10.72 in Tbr1 animals, *P*=0.002). By contrast, cell death was negligible and similar in both conditions (Fig. 1J). Together, these findings suggest that Tbr1 misexpression during embryonic development alters the migration and final position of the neurons derived from NPCs.

We then used a marker of the cell cycle, an antibody against Ki67, to check whether Tbr1 misexpression affects the number of actively cycling NPCs [58,59]. No difference in the proportion of Ki67^+^ cells was observed when electroporation was performed at E14.5 and the embryos were analysed at E18.5 (data not shown). Indeed, when this assay was performed over a narrower time-window, analysing the cerebral cortex of E14.5 electroporated embryos 24 hours later (E15.5 embryos), GFP^+^ cells were detected in both conditions (Fig. 2). Moreover, in the embryos that received the pCAG-Tbr1-EGFP construct, nearly 100% of these GFP^+^ cells also expressed Tbr1 whereas GFP^+^-Tbr1^+^ cells were not found in the control condition (Fig. 2A´-B, G). Sections from these E15.5 embryos were then immunostained with antibodies against Ki67 and Tbr2, a TF widely expressed by IPs in the developing cerebral cortex [1,60]. No significant differences in the percentages of GFP^+^ cells positive for Ki67 (*P*=0.807: Fig. 2C´-D) or Tbr2 were evident between the two conditions (*P*=0.553: Fig. 2E´-F). Thus, it appears that Tbr1 overexpression does not affect the number of cycling progenitors nor that of the IPs. To further explore whether proliferation is altered, pregnant mothers received IdU 3 hours after electroporation and CIdU 24 hours after electroporation. At E18.5, the percentages of double labelled GFP^+^IdU^+^ and GFP^+^CIdU^+^ cells, as well as that of triple labelled GFP^+^IdU^+^CIdU^+^ cells were similar in both conditions, although only a small number of animals could be analysed (Supplementary Fig. S1); hence Tbr1 misexpression appears not to alter the number of cells in S-phase nor that of cells staying in cell cycle from E14.5 to E18.5. In addition, Tbr1 misexpression did not significantly change the percentage of GFP^+^TuJ1^+^/GFP^+^ cells at E18.5 (data not shown) and almost 100% of GFP^+^ cells were NeuN^+^ at P7, both under control and Tbr1 misexpression conditions (Supplementary Fig. S2). These data suggest that neurogenesis was not affected by Tbr1 misexpression.

**Tbr1 plays a role regulating neuronal polarization and radial cell migration**

Since Tbr1 misexpression alters the distribution of neurons in the developing neocortex (Fig. 1), an ectopic increase in Tbr1 levels is likely to inhibit the migration of newly formed neurons. As such, we analysed neuronal polarization, given that immature projection neurons modify their morphology from multipolar to bipolar when crossing the IZ, becoming radially orientated [5-7,61,62]. Tbr1 misexpression significantly increased the proportion of multipolar neurons in the IZ (15.93% ± 1.17 in control, 26.60% ± 0.21 in Tbr1 overexpressing mice, *P*=0.009) and in layers V-VI (0.62% ± 0.26 in control, 2.31% ± 0.68 in Tbr1 overexpressing mice, *P*=0.032 (Fig. 3A-F, I). Furthermore, it increased the percentage of non-radial bipolar neurons in layers V-VI (1.34% ± 0.43 in control, 4.50% ± 0.65 in Tbr1 overexpressing mice, *P*=0.002: Fig. 3G, J) and decreased the percentage of radial bipolar neurons (98.03% ± 0.56 in control, 93.17% ± 0.97 in Tbr1 overexpressing mice, *P*=0.001: Fig. 3H, K). These data suggest that sustained Tbr1 expression in part disrupted neuronal migration by altering the correct transition from a multipolar to a radial bipolar state, which could slow their migration along radial glia. However, Tbr1 misexpression did not appear to change the radial glia scaffold (Supplementary Fig. S3) when it was analysed by immunostaining with an RC2 antibody that is a marker of radial glial [63].

**Tbr1 misexpression alters neuron subtype specification in the neocortex**

In addition to producing defects in cell migration, Tbr1 misexpression might also affect the molecular identity of relevant neuronal subtypes. To assess this, we analysed embryos electroporated at E14.5 and then immunostained at E18.5 sections with antibodies against: Cux1, a marker of layer II-IV neurons [64]; CTIP2, a marker of layer V neurons [30,57]; and Sox5, a marker of layer VI-V neurons [65] (Fig. 4A´-F). Of the three neuronal subtypes studied here, Cux1^+^ neurons were those generated most abundantly between days E14.5-E18.5 in the embryos electroporated with the control pCAG-EGFP construct (31.86% as opposed to 5.38% CTIP2^+^ cells and 4.99% Sox5^+^ cells (Fig. 4G,H,I). However, Tbr1 misexpression significantly decreased the proportion of Cux1^+^-GFP^+^ cells in layers II-IV (1.7-fold or 41.4%, *P*=0.006: Fig. 4A´-B, G) and that of CTIP2^+^-GFP^+^ cells in layer V (2.4-fold or 58.0%, *P*=0.031: Fig. 4C´-D, H), resulting in 18.74% Cux1^+^ and 2.24% CTIP2^+^ cells. By contrast, Tbr1 misexpression increased the percentage of Sox5^+^-GFP^+^ cells in layers V-VI (5.2-fold or 520%, *P*=0.010: Fig. 4E´-F, I), resulting in 25.95% Sox5^+^ cells. The overall proportion of GFP^+^ cells expressing these three markers was 42.23% in the controls and 46.93% following Tbr1 overexpression. Hence, an increase in Tbr1 expression at these embryonic stages alters neuronal subtype specification and cerebral cortex lamination.

**Dendrite and axon growth is also affected by Tbr1 misexpression**

We next studied whether ectopic Tbr1 overexpression affects dendrite and axon development in the neocortex. As such, we electroporated embryos at E14.5 and analysed the dendrite length and number at E18.5, as well as the callosal axon tract. We first observed that Tbr1 misexpression significantly decreased the length and the number of dendrites on layer II-III neurons (28.4%, *P*=0.001 and 59.2%, *P*=0.001, respectively: Fig. 5A-D). When the *corpus callosum* was analysed Tbr1 overexpression significantly reduced callosal axon fibre length (44.07%, *P*=0.008: Fig. 5E-G) and thickness (58.06%, *P*=0.003: Fig. 5H), even though the total number of GFP electroporated cells was similar in both groups (*P*=0.248; Fig. 5I). Thus, sustained Tbr1 misexpression impairs dendritogenesis, as well as callosal axon growth and guidance.

**Effect of Tbr1 misexpression in postnatal neocortical development**

We next asked if the phenotypic alterations observed in Tbr1 electroporated embryos were maintained in postnatal animals, analysing P7 animals that were electroporated at E14.5. While at the developmental stages studied previously (E15.5 and E18.5) the proportion of GFP^+^-Tbr1^+^ cells relative to the GFP^+^ cells was close to 100% in P7 pCAG-Tbr1-EGFP electroporated mice, very few double labelled cells were detected in the control (pCAG-EGFP) animals (*P*=0.0001: Fig. 6A-C). It is important to note that at P7 Tbr1 expression it is not restricted to layer VI and layer V neurons but it is also mildly expressed in upper layer neurons (Supplementary Fig. S4). We studied the impact of sustained Tbr1 expression on dendrites and found that it produced a significant reduction in the number of basal dendrites (including dendrite branches) per neuron in layers II-III (44.8%, *P*=0.0001: Fig. 6D-F), as well as a reduction in dendrite length (53.3%, *P*=0.015: Fig. 6G) and in the area occupied by the dendrites (53.22%, *P*=0.015: Fig. 6H). These results indicate that Tbr1 misexpression alters dendrite growth and number, and that this effect persists in postnatal animals.

To determine the distribution of GFP^+^ cells in the different layers of the cortex, P7 sections were stained with antibodies to Cux1 in order to clearly distinguish GFP^+^ cells in layer I, from the upper (II-IV) and deep layers (V-VI: Fig. 6I-J). By P7, the vast majority of GFP^+^ cells were located in upper layers II-IV and labelled neurons were virtually absent from layers V-VI in the control mice. However, following Tbr1 overexpression 78% and 10% of GFP^+^ cells were detected in layers II-IV and layers V-VI, respectively, although these changes were not statistically significant (Fig. 6L). The percentages of GFP^+^Cux1^+^ cells were similar in both conditions (94.97% ± 0.7219 and 91.43 % ± 2.554 in control and Tbr1 overexpressing animals, respectively) (Fig. 6K). By contrast, when cells that were immunostained with GFP and Sox5 antibodies were counted in sections from P7 mice, there was significantly higher proportions of GFP^+^Sox5^+^ cells in layers II-III (70%, P = 0.013, Fig. 7A-C) and in layers IV-V (76.6%, P = 0.0095) and a higher proportion of double positive cells in layer VI (37%, P = 0.050, Fig. 7A-B, D) following Tbr1 misexpression. All these findings suggest that the defects in migration provoked by Tbr1 misexpression were not evident in P7 animals, although the alterations to the specification of Sox5 expressing neurons persisted. In addition, orientation defects in the targeted neurons that overexpress Tbr1 in lower layers can be observed in Figs. 6J and 7B´-B.

**DISCUSSION**

Loss-of-function studies have shown that Tbr1 is critical for the differentiation of Cajal-Retzius, subplate and layer VI neurons, and for both neuron and laminar specification, as well as axon pathfinding [12,25-31]. However, there is little evidence of the effects of ectopic Tbr1 overexpression, a fact that could be relevant to understand the aetiopathology of some ASD cases [52]. Here we tested the impact of Tbr1 misexpression on NPCs and found defects in the development of glutamatergic projection neurons in the neocortex.

From previous studies [2,6,8] and following the electroporation of our control pCAG-EGFP plasmid, at E14.5 the vast majority of layer V (CTIP^+^) and layer VI (Tbr1^+^ and Sox5^+^) neurons have already been born, whereas layer II-IV neurons are still being generated. Thus, here Tbr1 was misexpressed in NPCs that are mostly destined to produce layer II-IV projection neurons, which disturbed the correct pattern of neuronal migration, neuron subtype specification, as well as dendrite development and that of the callosal axon tract. By contrast, cycling cells, the number of Tbr2^+^ progenitors and cell death were not significantly affected by Tbr1 misexpression in this manner. Tbr1 overexpressing cells accumulate in the IZ, and in layers VI and V, such that fewer neurons reach layers II-IV at E18.5. This defect in neuron migration to superficial layers under our conditions is somewhat similar to that reported for parvalbumin interneurons in a mutant mouse expressing higher levels of Tbr1 in the brain, although this effect could be secondary to changes that also affect projection neurons [52,53,55].

Our findings indicate significant increases in the proportion of immature multipolar neurons in both the IZ and layers V-VI, and that of non-radial bipolar neurons in layers V-VI, while the proportion of radial bipolar neurons decreases. These changes potentially explain the delay in neuronal migration caused by Tbr1 misexpression, best illustrated by the reduction of Cux1^+^ neurons in layers II-IV at E18.5 that it is not observed at P7. In fact, the transition from a multipolar to radial morphology of migrating neocortical neurons is critical for their migration along radial glial fibres in order to reach the cortical plate [5,6,61]. Although not studied here, it is tempting to speculate that Tbr1 misexpression could alter its association with CASK, which fulfils an important role during neuronal migration and morphological differentiation, partially due to the activation of reelin transcription by Tbr1 [12,33,34,42,66,67]. Furthermore, our results show that the morphological alterations caused by Tbr1 misexpression not only affect migrating neurons but also, they are observed in dendrites of neurons reaching layers II-III, as well as in callosal axons. These latter results suggest that Tbr1 upregulation could affect neuronal morphology, possibly by acting on genes that regulate cytoskeletal dynamics, neurite outgrowth and fasciculation [32,35,68]. The smaller number of neurons reaching layers II-IV might also explain the reduced thickness and length of the callosal axon tract in E18.5 brains following Tbr1 misexpression. The disturbance of migration at E18.5 is not significant at P7, suggesting a dampening of the effect of Tbr1, although dendrite morphology is still affected at this stage P7 such that some effects of Tbr1 misexpression appear to persist. Thus, the alterations to dendrites could have a negative effect on the connectivity of mature neurons, consequently affecting cortical networks.

The changes in neuronal lamination triggered by Tbr1 misexpression coincide with a decrease in the proportion of cells expressing molecular markers typical of layers II-IV (Cux1) and layer V (CTIP), and with an increase in the percentage of Sox5^+^ cells in layers VI-V at E18.5. Our results fit well with the phenotype found in the developing neocortex of Tbr1 null mutant mice in which the expression of layer II-IV markers increases, while that of layer VI markers diminishes [26-28]. Interestingly, our results are also consistent with the callosal defects reported in the Tbr1 null E18.5 mouse [12] and they support the concept that Tbr1 levels must be tightly regulated, both temporally and spatially, for the correct development of the cerebral cortex layers and the *corpus callosum*. The increase in the percentage of cells that accumulate in layer V when Tbr1 levels are sustained, concomitant with a decrease in the proportion of CTIP^+^ neurons, suggest that Tbr1 might partially repress CTIP, as reported previously [27,30]. However, the increase in the number of Sox5^+^ neurons at P7 in the upper layers, when the defects in migration are less prominent, indicates that disruptions to the specification of these neurons is probably permanent. If so, the cortical upper layer will contain an imbalance in neuronal subtypes, as indicated by the similar numbers of Cux1^+^ cells and the larger number of Sox5^+^ cells at P7. Notably, it has been proposed that the dysregulation of specific genes in upper-layer projection neurons, including SOX5, correlates with the clinical severity of ASD [77].

As mentioned above, mutations and microdeletions that cause TBR1 loss-of-function in humans are associated with cerebral cortex malformations that are accompanied by ASD and intellectual disability [33,36,44,47,48,50,51,69]. This emphasizes the importance of TBR1 in human brain development and function. Indeed, *Tbr1^+/−^* heterozygous mice display autism-like phenotypes, such as impaired social interactions, abnormal ultrasonic vocalization, and defects in associative memory and cognitive flexibility [68,70]. However, increased brain TBR1 levels have also recently been associated with altered synaptic transmission, ASD-like deficits and savant syndrome [52,53,55]. Importantly, neurons carrying the CHD7 intronic variant identified in ASD individuals displayed morphological defects that were rescued by downregulating TBR1, suggesting a causal effect of TBR1 upregulation in the defects reported [53]. Nonetheless, it will be necessary to perform additional studies to elucidate whether the deficits reported in these recent studies are a direct consequence of TBR1 upregulation.

Although our experimental strategy does not reproduce a genetic mutation that mimics the patient’s genome, our findings suggest that Tbr1 misexpression either delays or permanently disrupts neocortical neuronal migration, subtype specification, dendrite morphology and callosal axon formation. In fact, the abnormalities in neuron migration and in the development of callosal neuron processes in upper layer neurons detected here are compatible with the cortical-cortical connectivity failures observed in ASD individuals [33,47,71-75]. Moreover, our finding that neurons accumulate in the IZ is also compatible with the subcortical heterotopia characteristic of ASD brains [76].

In conclusion, together with previous results, our findings support the concept that Tbr1 levels must be finely regulated, both temporally and spatially, to prevent the occurrence of the brain malformations that underlie ASD and intellectual disability.

**Author contributions**

I.C.: Experimental design, collection and assembly of data, data analysis and interpretation, manuscript writing and revision. J.P.: Experimental design, collection and assembly of data, data analysis and interpretation, manuscript writing. V.K.: Experimental design, collection and assembly of data. H.R.M.-G.: Experimental design and collection of data. M.E.: Design of cloning strategies. M.J.R.: Collection of data. J.M.C.: Provision of study material, financial support. S.T.: Experimental design, data analysis and interpretation, financial support. C.V.: Study conception and experimental design, data analysis and interpretation, financial and administrative support, manuscript writing and final approval of manuscript.

**Statements and Declarations**

**Competing interest:** The authors have no potential conflicts of interest to declare.

**Compliance with Ethical Standards**

**Research involving Animals:** All animal care and handling was carried out in accordance with European Union guidelines (directive 2010/63/EU) and Spanish legislation (Law 32/2007 and RD 53/2013). The protocols were approved by the Ethics Committees of the Consejo Superior de Investigaciones Científicas (CSIC, Madrid, Spain) and the Comunidad de Madrid (Spain). Some experiments related to the data shown in Figure 1, as well as the Ki67 (at E18.5) and RC2 results, were performed at the Tata Institute of Fundamental Research following animal protocols approved by the Institution’s Animal Ethics Committee and according to regulations devised by the Committee for the Purpose of Control and Supervision of Experiments on Animals. Animals were administered food and water ad libitum, and they were housed in strictly controlled environmental conditions: 12 h light/dark cycle, 22 ºC temperature and 44% humidity. All efforts were made to ameliorate the animal´s suffering.

**Data, Material and/or Code availability and Authors’ contribution statements:** The data that support the findings of this study are available on reasonably request from the corresponding author.

**Acknowledgments and Funding**: We thank Carlos J Villacís and Dr Rebeca Vecino (Instituto Cajal-CSIC, Madrid, Spain) for their technical support in cloning and confocal imaging, respectively, Dr Juan J. Garrido (Instituto Cajal-CSIC, Madrid, Spain) for sharing his electroporation equipment with us, Dr M. Sefton (BiomedRed, Madrid, Spain) for English editing, Lucía Vicario (Instituto Cajal-CSIC, Madrid, Spain) for help in composing the figures, and the ‘Unidad de Imagen Científica y Microscopía’ (Instituto Cajal-CSIC, Madrid) for helping us with the morphological analyses. This work was funded by grants from the Spanish “Ministerio de Economía y Competitividad and Ministerio de Ciencia, Innovación y Universidades” (MINECO and MICIU: SAF2013-4759R and SAF2016-80419-R to C.V., RTI2018-099001-B-I00 to J.M.C), CIBERNED (CB06/05/0065 to C.V.), RETICS-Red de Terapia Celular (RD16/0011/0012 to J.M.C.), the “Comunidad de Madrid” (S2011/BMD-2336) and the CSIC Intramural program (Ref. 201320E054 to C.V.), the Generalitat de Catalunya (2017SGR-1408 to J.M.C.), and by a University Grants Commission fellowship to V.K. and Department of Atomic Energy, Government of India Project (RTI4003 to S.T.).

**Consent to participate:** Not applicable

**Consent to publish:** Not applicable

**REFERENCE LIST**

1. Englund C, Fink A, Lau C, Pham D, Daza RA, Bulfone A, Kowalczyk T, Hevner RF (2005) Pax6, Tbr2, and Tbr1 are expressed sequentially by radial glia, intermediate progenitor cells, and postmitotic neurons in developing neocortex. J Neurosci 25 (1):247-251. doi:10.1523/JNEUROSCI.2899-04.2005

2. Greig LC, Woodworth MB, Galazo MJ, Padmanabhan H, Macklis JD (2013) Molecular logic of neocortical projection neuron specification, development and diversity. Nat Rev Neurosci 14 (11):755-769. doi:10.1038/nrn3586

3. Kowalczyk T, Pontious A, Englund C, Daza RA, Bedogni F, Hodge R, Attardo A, Bell C, Huttner WB, Hevner RF (2009) Intermediate neuronal progenitors (basal progenitors) produce pyramidal-projection neurons for all layers of cerebral cortex. Cereb Cortex 19 (10):2439-2450. doi:10.1093/cercor/bhn260

4. Xing L, Wilsch-Brauninger M, Huttner WB (2021) How neural stem cells contribute to neocortex development. Biochem Soc Trans 49 (5):1997-2006. doi:10.1042/BST20200923

5. Kriegstein AR, Noctor SC (2004) Patterns of neuronal migration in the embryonic cortex. Trends Neurosci 27 (7):392-399. doi:10.1016/j.tins.2004.05.001

6. Barnes AP, Polleux F (2009) Establishment of axon-dendrite polarity in developing neurons. Annu Rev Neurosci 32:347-381. doi:10.1146/annurev.neuro.31.060407.125536

7. Cooper JA (2014) Molecules and mechanisms that regulate multipolar migration in the intermediate zone. Front Cell Neurosci 8:386. doi:10.3389/fncel.2014.00386

8. Molyneaux BJ, Arlotta P, Menezes JR, Macklis JD (2007) Neuronal subtype specification in the cerebral cortex. Nat Rev Neurosci 8 (6):427-437. doi:10.1038/nrn2151

9. Matho KS, Huilgol D, Galbavy W, He M, Kim G, An X, Lu J, Wu P, Di Bella DJ, Shetty AS, Palaniswamy R, Hatfield J, Raudales R, Narasimhan A, Gamache E, Levine JM, Tucciarone J, Szelenyi E, Harris JA, Mitra PP, Osten P, Arlotta P, Huang ZJ (2021) Genetic dissection of the glutamatergic neuron system in cerebral cortex. Nature 598 (7879):182-187. doi:10.1038/s41586-021-03955-9

10. Bulfone A, Smiga SM, Shimamura K, Peterson A, Puelles L, Rubenstein JL (1995) T-brain-1: a homolog of Brachyury whose expression defines molecularly distinct domains within the cerebral cortex. Neuron 15 (1):63-78. doi:10.1016/0896-6273(95)90065-9

11. Bulfone A, Wang F, Hevner R, Anderson S, Cutforth T, Chen S, Meneses J, Pedersen R, Axel R, Rubenstein JL (1998) An olfactory sensory map develops in the absence of normal projection neurons or GABAergic interneurons. Neuron 21 (6):1273-1282. doi:10.1016/s0896-6273(00)80647-9

12. Hevner RF, Shi L, Justice N, Hsueh Y, Sheng M, Smiga S, Bulfone A, Goffinet AM, Campagnoni AT, Rubenstein JL (2001) Tbr1 regulates differentiation of the preplate and layer 6. Neuron 29 (2):353-366. doi:10.1016/s0896-6273(01)00211-2

13. Brox A, Puelles L, Ferreiro B, Medina L (2004) Expression of the genes Emx1, Tbr1, and Eomes (Tbr2) in the telencephalon of Xenopus laevis confirms the existence of a ventral pallial division in all tetrapods. J Comp Neurol 474 (4):562-577. doi:10.1002/cne.20152

14. Saito T, Hanai S, Takashima S, Nakagawa E, Okazaki S, Inoue T, Miyata R, Hoshino K, Akashi T, Sasaki M, Goto Y, Hayashi M, Itoh M (2011) Neocortical layer formation of human developing brains and lissencephalies: consideration of layer-specific marker expression. Cereb Cortex 21 (3):588-596. doi:10.1093/cercor/bhq125

15. Imamura F, Greer CA (2013) Pax6 regulates Tbr1 and Tbr2 expressions in olfactory bulb mitral cells. Mol Cell Neurosci 54:58-70. doi:10.1016/j.mcn.2013.01.002

16. Takashima Y, Suzuki A (2013) Regulation of organogenesis and stem cell properties by T-box transcription factors. Cell Mol Life Sci 70 (20):3929-3945. doi:10.1007/s00018-013-1305-5

17. Papaioannou VE (2014) The T-box gene family: emerging roles in development, stem cells and cancer. Development 141 (20):3819-3833. doi:10.1242/dev.104471

18. Cipriani S, Nardelli J, Verney C, Delezoide AL, Guimiot F, Gressens P, Adle-Biassette H (2016) Dynamic Expression Patterns of Progenitor and Pyramidal Neuron Layer Markers in the Developing Human Hippocampus. Cereb Cortex 26 (3):1255-1271. doi:10.1093/cercor/bhv079

19. Frese CK, Mikhaylova M, Stucchi R, Gautier V, Liu Q, Mohammed S, Heck AJR, Altelaar AFM, Hoogenraad CC (2017) Quantitative Map of Proteome Dynamics during Neuronal Differentiation. Cell Rep 18 (6):1527-1542. doi:10.1016/j.celrep.2017.01.025

20. Telley L, Govindan S, Prados J, Stevant I, Nef S, Dermitzakis E, Dayer A, Jabaudon D (2016) Sequential transcriptional waves direct the differentiation of newborn neurons in the mouse neocortex. Science 351 (6280):1443-1446. doi:10.1126/science.aad8361

21. Mihalas AB, Hevner RF (2017) Control of Neuronal Development by T-Box Genes in the Brain. Curr Top Dev Biol 122:279-312. doi:10.1016/bs.ctdb.2016.08.001

22. Docampo-Seara A, Lanoizelet M, Lagadec R, Mazan S, Candal E, Rodriguez MA (2019) Mitral cell development in the olfactory bulb of sharks: evidences of a conserved pattern of glutamatergic neurogenesis. Brain Struct Funct 224 (7):2325-2341. doi:10.1007/s00429-019-01906-9

23. Mendez-Gomez HR, Vergano-Vera E, Abad JL, Bulfone A, Moratalla R, de Pablo F, Vicario-Abejon C (2011) The T-box brain 1 (Tbr1) transcription factor inhibits astrocyte formation in the olfactory bulb and regulates neural stem cell fate. Mol Cell Neurosci 46 (1):108-121. doi:10.1016/j.mcn.2010.08.011

24. Diaz-Guerra E, Pignatelli J, Nieto-Estevez V, Vicario-Abejon C (2013) Transcriptional regulation of olfactory bulb neurogenesis. Anat Rec (Hoboken) 296 (9):1364-1382. doi:10.1002/ar.22733

25. Hevner RF, Miyashita-Lin E, Rubenstein JL (2002) Cortical and thalamic axon pathfinding defects in Tbr1, Gbx2, and Pax6 mutant mice: evidence that cortical and thalamic axons interact and guide each other. J Comp Neurol 447 (1):8-17. doi:10.1002/cne.10219

26. Bedogni F, Hodge RD, Elsen GE, Nelson BR, Daza RA, Beyer RP, Bammler TK, Rubenstein JL, Hevner RF (2010) Tbr1 regulates regional and laminar identity of postmitotic neurons in developing neocortex. Proc Natl Acad Sci U S A 107 (29):13129-13134. doi:10.1073/pnas.1002285107

27. McKenna WL, Betancourt J, Larkin KA, Abrams B, Guo C, Rubenstein JL, Chen B (2011) Tbr1 and Fezf2 regulate alternate corticofugal neuronal identities during neocortical development. J Neurosci 31 (2):549-564. doi:10.1523/JNEUROSCI.4131-10.2011

28. Han W, Kwan KY, Shim S, Lam MM, Shin Y, Xu X, Zhu Y, Li M, Sestan N (2011) TBR1 directly represses Fezf2 to control the laminar origin and development of the corticospinal tract. Proc Natl Acad Sci U S A 108 (7):3041-3046. doi:10.1073/pnas.1016723108

29. Fazel Darbandi S, Robinson Schwartz SE, Qi Q, Catta-Preta R, Pai EL, Mandell JD, Everitt A, Rubin A, Krasnoff RA, Katzman S, Tastad D, Nord AS, Willsey AJ, Chen B, State MW, Sohal VS, Rubenstein JLR (2018) Neonatal Tbr1 Dosage Controls Cortical Layer 6 Connectivity. Neuron 100 (4):831-845 e837. doi:10.1016/j.neuron.2018.09.027

30. Canovas J, Berndt FA, Sepulveda H, Aguilar R, Veloso FA, Montecino M, Oliva C, Maass JC, Sierralta J, Kukuljan M (2015) The Specification of Cortical Subcerebral Projection Neurons Depends on the Direct Repression of TBR1 by CTIP1/BCL11a. J Neurosci 35 (19):7552-7564. doi:10.1523/JNEUROSCI.0169-15.2015

31. Srinivasan K, Leone DP, Bateson RK, Dobreva G, Kohwi Y, Kohwi-Shigematsu T, Grosschedl R, McConnell SK (2012) A network of genetic repression and derepression specifies projection fates in the developing neocortex. Proc Natl Acad Sci U S A 109 (47):19071-19078. doi:10.1073/pnas.1216793109

32. Fazel Darbandi S, Robinson Schwartz SE, Pai EL, Everitt A, Turner ML, Cheyette BNR, Willsey AJ, State MW, Sohal VS, Rubenstein JLR (2020) Enhancing WNT Signaling Restores Cortical Neuronal Spine Maturation and Synaptogenesis in Tbr1 Mutants. Cell Rep 31 (2):107495. doi:10.1016/j.celrep.2020.03.059

33. Najm J, Horn D, Wimplinger I, Golden JA, Chizhikov VV, Sudi J, Christian SL, Ullmann R, Kuechler A, Haas CA, Flubacher A, Charnas LR, Uyanik G, Frank U, Klopocki E, Dobyns WB, Kutsche K (2008) Mutations of CASK cause an X-linked brain malformation phenotype with microcephaly and hypoplasia of the brainstem and cerebellum. Nat Genet 40 (9):1065-1067. doi:10.1038/ng.194

34. Hsueh YP, Wang TF, Yang FC, Sheng M (2000) Nuclear translocation and transcription regulation by the membrane-associated guanylate kinase CASK/LIN-2. Nature 404 (6775):298-302. doi:10.1038/35005118

35. Huang TN, Hsueh YP (2015) Brain-specific transcriptional regulator T-brain-1 controls brain wiring and neuronal activity in autism spectrum disorders. Front Neurosci 9:406. doi:10.3389/fnins.2015.00406

36. Willsey AJ, Sanders SJ, Li M, Dong S, Tebbenkamp AT, Muhle RA, Reilly SK, Lin L, Fertuzinhos S, Miller JA, Murtha MT, Bichsel C, Niu W, Cotney J, Ercan-Sencicek AG, Gockley J, Gupta AR, Han W, He X, Hoffman EJ, Klei L, Lei J, Liu W, Liu L, Lu C, Xu X, Zhu Y, Mane SM, Lein ES, Wei L, Noonan JP, Roeder K, Devlin B, Sestan N, State MW (2013) Coexpression networks implicate human midfetal deep cortical projection neurons in the pathogenesis of autism. Cell 155 (5):997-1007. doi:10.1016/j.cell.2013.10.020

37. O'Roak BJ, Vives L, Fu W, Egertson JD, Stanaway IB, Phelps IG, Carvill G, Kumar A, Lee C, Ankenman K, Munson J, Hiatt JB, Turner EH, Levy R, O'Day DR, Krumm N, Coe BP, Martin BK, Borenstein E, Nickerson DA, Mefford HC, Doherty D, Akey JM, Bernier R, Eichler EE, Shendure J (2012) Multiplex targeted sequencing identifies recurrently mutated genes in autism spectrum disorders. Science 338 (6114):1619-1622. doi:10.1126/science.1227764

38. Traylor RN, Dobyns WB, Rosenfeld JA, Wheeler P, Spence JE, Bandholz AM, Bawle EV, Carmany EP, Powell CM, Hudson B, Schultz RA, Shaffer LG, Ballif BC (2012) Investigation of TBR1 Hemizygosity: Four Individuals with 2q24 Microdeletions. Mol Syndromol 3 (3):102-112. doi:10.1159/000342008

39. Burrage LC, Eble TN, Hixson PM, Roney EK, Cheung SW, Franco LM (2013) A mosaic 2q24.2 deletion narrows the critical region to a 0.4 Mb interval that includes TBR1, TANK, and PSMD14. Am J Med Genet A 161A (4):841-844. doi:10.1002/ajmg.a.35751

40. Palumbo O, Fichera M, Palumbo P, Rizzo R, Mazzolla E, Cocuzza DM, Carella M, Mattina T (2014) TBR1 is the candidate gene for intellectual disability in patients with a 2q24.2 interstitial deletion. Am J Med Genet A 164A (3):828-833. doi:10.1002/ajmg.a.36363

41. Hamdan FF, Srour M, Capo-Chichi JM, Daoud H, Nassif C, Patry L, Massicotte C, Ambalavanan A, Spiegelman D, Diallo O, Henrion E, Dionne-Laporte A, Fougerat A, Pshezhetsky AV, Venkateswaran S, Rouleau GA, Michaud JL (2014) De novo mutations in moderate or severe intellectual disability. PLoS Genet 10 (10):e1004772. doi:10.1371/journal.pgen.1004772

42. Deriziotis P, O'Roak BJ, Graham SA, Estruch SB, Dimitropoulou D, Bernier RA, Gerdts J, Shendure J, Eichler EE, Fisher SE (2014) De novo TBR1 mutations in sporadic autism disrupt protein functions. Nat Commun 5:4954. doi:10.1038/ncomms5954

43. Belengeanu V, Gamage TH, Farcas S, Stoian M, Andreescu N, Belengeanu A, Frengen E, Misceo D (2014) A de novo 2.3 Mb deletion in 2q24.2q24.3 in a 20-month-old developmentally delayed girl. Gene 539 (1):168-172. doi:10.1016/j.gene.2014.01.060

44. Chuang HC, Huang TN, Hsueh YP (2015) T-Brain-1--A Potential Master Regulator in Autism Spectrum Disorders. Autism Res 8 (4):412-426. doi:10.1002/aur.1456

45. Ernst C (2016) Proliferation and Differentiation Deficits are a Major Convergence Point for Neurodevelopmental Disorders. Trends Neurosci 39 (5):290-299. doi:10.1016/j.tins.2016.03.001

46. Notwell JH, Heavner WE, Darbandi SF, Katzman S, McKenna WL, Ortiz-Londono CF, Tastad D, Eckler MJ, Rubenstein JL, McConnell SK, Chen B, Bejerano G (2016) TBR1 regulates autism risk genes in the developing neocortex. Genome Res 26 (8):1013-1022. doi:10.1101/gr.203612.115

47. Vegas N, Cavallin M, Kleefstra T, de Boer L, Philbert M, Maillard C, Boddaert N, Munnich A, Hubert L, Bery A, Besmond C, Bahi-Buisson N (2018) Mutations in TBR1 gene leads to cortical malformations and intellectual disability. Eur J Med Genet 61 (12):759-764. doi:10.1016/j.ejmg.2018.09.012

48. McDermott JH, Study DDD, Clayton-Smith J, Briggs TA (2018) The TBR1-related autistic-spectrum-disorder phenotype and its clinical spectrum. Eur J Med Genet 61 (5):253-256. doi:10.1016/j.ejmg.2017.12.009

49. den Hoed J, Sollis E, Venselaar H, Estruch SB, Deriziotis P, Fisher SE (2018) Functional characterization of TBR1 variants in neurodevelopmental disorder. Sci Rep 8 (1):14279. doi:10.1038/s41598-018-32053-6

50. Kim Y, An JY (2020) Spatio-Temporal Roles of ASD-Associated Variants in Human Brain Development. Genes (Basel) 11 (5). doi:10.3390/genes11050535

51. Nambot S, Faivre L, Mirzaa G, Thevenon J, Bruel AL, Mosca-Boidron AL, Masurel-Paulet A, Goldenberg A, Le Meur N, Charollais A, Mignot C, Petit F, Rossi M, Metreau J, Layet V, Amram D, Boute-Benejean O, Bhoj E, Cousin MA, Kruisselbrink TM, Lanpher BC, Klee EW, Fiala E, Grange DK, Meschino WS, Hiatt SM, Cooper GM, Olivie H, Smith WE, Dumas M, Lehman A, Study C, Inglese C, Nizon M, Guerrini R, Vetro A, Kaplan ES, Miramar D, Van Gils J, Fergelot P, Bodamer O, Herkert JC, Pajusalu S, Ounap K, Filiano JJ, Smol T, Piton A, Gerard B, Chantot-Bastaraud S, Bienvenu T, Li D, Juusola J, Devriendt K, Bilan F, Poe C, Chevarin M, Jouan T, Tisserant E, Riviere JB, Tran Mau-Them F, Philippe C, Duffourd Y, Dobyns WB, Hevner R, Thauvin-Robinet C (2020) De novo TBR1 variants cause a neurocognitive phenotype with ID and autistic traits: report of 25 new individuals and review of the literature. Eur J Hum Genet 28 (6):770-782. doi:10.1038/s41431-020-0571-6

52. Yook C, Kim K, Kim D, Kang H, Kim SG, Kim E, Kim SY (2019) A TBR1-K228E Mutation Induces Tbr1 Upregulation, Altered Cortical Distribution of Interneurons, Increased Inhibitory Synaptic Transmission, and Autistic-Like Behavioral Deficits in Mice. Front Mol Neurosci 12:241. doi:10.3389/fnmol.2019.00241

53. Song J, Yang X, Zhou Y, Chen L, Zhang X, Liu Z, Niu W, Zhan N, Fan X, Khan AA, Kuang Y, Song L, He G, Li W (2019) Dysregulation of neuron differentiation in an autistic savant with exceptional memory. Mol Brain 12 (1):91. doi:10.1186/s13041-019-0507-7

54. Hughes JEA, Ward J, Gruffydd E, Baron-Cohen S, Smith P, Allison C, Simner J (2018) Savant syndrome has a distinct psychological profile in autism. Mol Autism 9:53. doi:10.1186/s13229-018-0237-1

55. Zhang R, He H, Yuan B, Wu Z, Wang X, Du Y, Chen Y, Qiu Z (2021) An Intronic Variant of CHD7 Identified in Autism Patients Interferes with Neuronal Differentiation and Development. Neurosci Bull 37 (8):1091-1106. doi:10.1007/s12264-021-00685-w

56. Niwa H, Yamamura K, Miyazaki J (1991) Efficient selection for high-expression transfectants with a novel eukaryotic vector. Gene 108 (2):193-199. doi:10.1016/0378-1119(91)90434-d

57. Arlotta P, Molyneaux BJ, Chen J, Inoue J, Kominami R, Macklis JD (2005) Neuronal subtype-specific genes that control corticospinal motor neuron development in vivo. Neuron 45 (2):207-221. doi:10.1016/j.neuron.2004.12.036

58. von Bohlen und Halbach O (2011) Immunohistological markers for proliferative events, gliogenesis, and neurogenesis within the adult hippocampus. Cell Tissue Res 345 (1):1-19. doi:10.1007/s00441-011-1196-4

59. Mendez-Gomez HR, Vicario-Abejon C (2012) The homeobox gene Gsx2 regulates the self-renewal and differentiation of neural stem cells and the cell fate of postnatal progenitors. PLoS One 7 (1):e29799. doi:10.1371/journal.pone.0029799

60. Sessa A, Mao CA, Hadjantonakis AK, Klein WH, Broccoli V (2008) Tbr2 directs conversion of radial glia into basal precursors and guides neuronal amplification by indirect neurogenesis in the developing neocortex. Neuron 60 (1):56-69. doi:10.1016/j.neuron.2008.09.028

61. LoTurco JJ, Bai J (2006) The multipolar stage and disruptions in neuronal migration. Trends Neurosci 29 (7):407-413. doi:10.1016/j.tins.2006.05.006

62. Ohshima T, Hirasawa M, Tabata H, Mutoh T, Adachi T, Suzuki H, Saruta K, Iwasato T, Itohara S, Hashimoto M, Nakajima K, Ogawa M, Kulkarni AB, Mikoshiba K (2007) Cdk5 is required for multipolar-to-bipolar transition during radial neuronal migration and proper dendrite development of pyramidal neurons in the cerebral cortex. Development 134 (12):2273-2282. doi:10.1242/dev.02854

63. Campbell K, Gotz M (2002) Radial glia: multi-purpose cells for vertebrate brain development. Trends Neurosci 25 (5):235-238. doi:10.1016/s0166-2236(02)02156-2

64. Nieto M, Monuki ES, Tang H, Imitola J, Haubst N, Khoury SJ, Cunningham J, Gotz M, Walsh CA (2004) Expression of Cux-1 and Cux-2 in the subventricular zone and upper layers II-IV of the cerebral cortex. J Comp Neurol 479 (2):168-180. doi:10.1002/cne.20322

65. Lai T, Jabaudon D, Molyneaux BJ, Azim E, Arlotta P, Menezes JR, Macklis JD (2008) SOX5 controls the sequential generation of distinct corticofugal neuron subtypes. Neuron 57 (2):232-247. doi:10.1016/j.neuron.2007.12.023

66. Wang GS, Hong CJ, Yen TY, Huang HY, Ou Y, Huang TN, Jung WG, Kuo TY, Sheng M, Wang TF, Hsueh YP (2004) Transcriptional modification by a CASK-interacting nucleosome assembly protein. Neuron 42 (1):113-128. doi:10.1016/s0896-6273(04)00139-4

67. Bock HH, May P (2016) Canonical and Non-canonical Reelin Signaling. Front Cell Neurosci 10:166. doi:10.3389/fncel.2016.00166

68. Huang TN, Chuang HC, Chou WH, Chen CY, Wang HF, Chou SJ, Hsueh YP (2014) Tbr1 haploinsufficiency impairs amygdalar axonal projections and results in cognitive abnormality. Nat Neurosci 17 (2):240-247. doi:10.1038/nn.3626

69. O'Roak BJ, Vives L, Girirajan S, Karakoc E, Krumm N, Coe BP, Levy R, Ko A, Lee C, Smith JD, Turner EH, Stanaway IB, Vernot B, Malig M, Baker C, Reilly B, Akey JM, Borenstein E, Rieder MJ, Nickerson DA, Bernier R, Shendure J, Eichler EE (2012) Sporadic autism exomes reveal a highly interconnected protein network of de novo mutations. Nature 485 (7397):246-250. doi:10.1038/nature10989

70. Lee EJ, Lee H, Huang TN, Chung C, Shin W, Kim K, Koh JY, Hsueh YP, Kim E (2015) Trans-synaptic zinc mobilization improves social interaction in two mouse models of autism through NMDAR activation. Nat Commun 6:7168. doi:10.1038/ncomms8168

71. Freitag CM, Luders E, Hulst HE, Narr KL, Thompson PM, Toga AW, Krick C, Konrad C (2009) Total brain volume and corpus callosum size in medication-naive adolescents and young adults with autism spectrum disorder. Biol Psychiatry 66 (4):316-319. doi:10.1016/j.biopsych.2009.03.011

72. Di Martino A, Yan CG, Li Q, Denio E, Castellanos FX, Alaerts K, Anderson JS, Assaf M, Bookheimer SY, Dapretto M, Deen B, Delmonte S, Dinstein I, Ertl-Wagner B, Fair DA, Gallagher L, Kennedy DP, Keown CL, Keysers C, Lainhart JE, Lord C, Luna B, Menon V, Minshew NJ, Monk CS, Mueller S, Muller RA, Nebel MB, Nigg JT, O'Hearn K, Pelphrey KA, Peltier SJ, Rudie JD, Sunaert S, Thioux M, Tyszka JM, Uddin LQ, Verhoeven JS, Wenderoth N, Wiggins JL, Mostofsky SH, Milham MP (2014) The autism brain imaging data exchange: towards a large-scale evaluation of the intrinsic brain architecture in autism. Mol Psychiatry 19 (6):659-667. doi:10.1038/mp.2013.78

73. Wegiel J, Kaczmarski W, Flory M, Martinez-Cerdeno V, Wisniewski T, Nowicki K, Kuchna I, Wegiel J (2018) Deficit of corpus callosum axons, reduced axon diameter and decreased area are markers of abnormal development of interhemispheric connections in autistic subjects. Acta Neuropathol Commun 6 (1):143. doi:10.1186/s40478-018-0645-7

74. Yao S, Becker B, Kendrick KM (2021) Reduced Inter-hemispheric Resting State Functional Connectivity and Its Association With Social Deficits in Autism. Front Psychiatry 12:629870. doi:10.3389/fpsyt.2021.629870

75. Prigge MBD, Lange N, Bigler ED, King JB, Dean DC, 3rd, Adluru N, Alexander AL, Lainhart JE, Zielinski BA (2021) A 16-year study of longitudinal volumetric brain development in males with autism. Neuroimage 236:118067. doi:10.1016/j.neuroimage.2021.118067

76. Wegiel J, Kuchna I, Nowicki K, Imaki H, Wegiel J, Marchi E, Ma SY, Chauhan A, Chauhan V, Bobrowicz TW, de Leon M, Louis LA, Cohen IL, London E, Brown WT, Wisniewski T (2010) The neuropathology of autism: defects of neurogenesis and neuronal migration, and dysplastic changes. Acta Neuropathol 119 (6):755-770. doi:10.1007/s00401-010-0655-4

77. Velmeshev D, Schirmer L, Jung D, Haeussler M, Perez Y, Mayer S, Bhaduri A, Goyal N, Rowitch DH, Kriegstein AR (2019) Single-cell genomics identifies cell type-specific molecular changes in autism. Science 364 (6441):685-689. doi:10.1126/science.aav8130

**FIGURE LEGENDS**

**Fig. 1** Tbr1 misexpression alters the distribution and positioning of migrating GFP^+^ cells in the cerebral cortex. A) Scheme of the constructs used in the experiments. Mouse embryos were electroporated *in utero* at E14.5, their brain was collected at E18.5 and vibratome sections were analysed by immunohistochemistry. A general view of the GFP^+^ cells in sagittal sections from the cerebral cortex of embryos electroporated with the pCAG-EGFP (B) or pCAG-Tbr1-EGFP plasmid (C). D) Quantification of the percentage of GFP^+^ cells in each zone and layer of the neocortex relative to the total number of GFP^+^ cells. E-G) Dual GFP and Tbr1 immunohistochemistry of coronal sections shows nearly 100% double positive cells in the pCAG-Tbr1-EGFP electroporated brains compared to a very low number of these cells in the pCAG-EGFP animals (***P<0.001; Student´s t test, n = 3 animals per condition). H-I) Dual GFP and CTIP2 immunohistochemistry allows the percentage of GFP^+^ cells in layers VI, V and II-IV to be quantified accurately. Tbr1 misexpression produced a significant accumulation of GFP^+^ cells in the IZ, and in layers VI and V, as well as a significant decrease of cells in layers II-IV. J) The percentage of cleaved caspase 3^+^ cells was very low, around 1% in both conditions (n = 3 animals per condition). The data in D and I are the mean ± S.E.M (n = 6-7 animals per condition): *P<0.05, **P<0.01, ***P<0.001 (Student´s t test). Scale bar, 500 μm (B, C), 100 μm (E, F, H). VZ, ventricular zone; SVZ, subventricular zone; IZ, Intermediate zone; SP, subplate.

**Fig. 2** Neither cycling cells nor the intermediate progenitor (IP) population are affected by Tbr1 misexpression. Mouse embryos were electroporated *in utero* at E14.5, their brain was collected at E15.5 and coronal vibratome sections were analysed by dual immunohistochemistry. A´-B, G) The efficiency of Tbr1 overexpression was nearly 100% in GFP^+^ cells from pCAG-Tbr1-EGFP electroporated mice, whereas no GFP and Tbr1 double labelled cells were detected in pCAG-EGFP electroporated mice. Tbr1 overexpression did not alter the percentage of GFP^+^Ki67^+^ cycling cells (C´-D, H) nor that of GFP^+^Tbr2^+^ IPs (E´-F, I). Examples of the double labelled cells are shown in the rectangles and boxes, and the data are the mean ± S.E.M. (n = 3-4 animals per condition). Scale bar, 20 μm.

**Fig. 3** The effect of Tbr1 misexpression on the morphology and orientation of migrating neurons. The images show migrating neurons in the cortex of E18.5 animals that were electroporated with the pCAG-EGFP (A, E) or pCAG-Tbr1-EGFP plasmid (B, F). The high magnification images show representative examples of multipolar (I), non-radial bipolar (J), and radial bipolar (K) GFP^+^ immature neurons from the IZ and layers V-VI of embryos electroporated with the pCAG-Tbr1-EGFP plasmid (neurons are indicated by rectangles in B and F). Vibratome sections were immunostained with an anti-GFP antibody and the quantification of the results is presented in the graphs: C (multipolar neurons in the IZ), D (multipolar neurons in layers V-VI), G (non-radial bipolar neurons in layers V-VI), and H (radial bipolar neurons in layers V-VI). Note the significant increase in the percentages of multipolar and non-radial bipolar neurons after Tbr1 overexpression. The data are the mean ± S.E.M (n=3 animals): *P<0.05, **P< 0.01, ***P< 0.001 (Student´s t test). Scale bar, 20 μm

**Fig. 4** Tbr1 misexpression alters neuronal specification and lamination. Coronal sections from E18.5 embryos electroporated with the pCAG-EGFP or pCAG-Tbr1-EGFP plasmid were dual immunostained with specific antibodies against GFP and Cux1 (A´-B), CTIP2 (C´-D) or Sox5 (E´-F). The cells marked by arrows are shown at a higher magnification in the insets. Tbr1 overexpression significantly reduced the percentage of Cux1^+^ cells and CTIP^+^ cells in layers II-IV and V, respectively, while it increased the percentage of Sox5^+^ cells in layers VI-V (G-I). The data are the mean ± S.E.M (n=3-5 animals): *P<0.05, ** P<0.01 (Student´s t test). Scale bar, 100 μm (A, B, D, E, G, H); 20 μm (insets).

**Fig. 5** Tbr1 misexpression impairs dendrite and callosal axon development. Coronal vibratome sections from electroporated embryos were immunostained at E18.5 with a GFP antibody and Hoechst was used to label the nuclei. Note that the complexity of neurons located in layer II-III (A-B) was markedly reduced in the embryos electroporated with pCAG-Tbr1-GFP, as indicated by the smaller dendrite length (P<0.001; C) and number of dendrites per neuron (P<0.001; D); Student´s t test, n = 15 neurons per condition). E-F) The images show axons forming the corpus callosum (CC) that cross to the contralateral hemisphere in the pCAG-EGFP electroporated animals, whereas this was markedly impaired following Tbr1 misexpression. The length and thickness of callosal fibers decreased significantly in the pCAG-Tbr1-EGFP electroporated animals (P<0.01; Student´s t test; G-H). The total number of GFP^+^ cells in the sections did not change (I). The data are the mean ± S.E.M (n = 3 animals per condition). Scale bar, 100 μm.

**Fig. 6** The altered dendrite morphology produced by Tbr1 misexpression is maintained in postnatal animals. Mouse embryos were electroporated *in utero* at E14.5, their brain was collected at P7 and vibratome sections were immunostained. A-C) Dual GFP and Tbr1 immunohistochemistry revealed nearly 100% of double positive cells following pCAG-Tbr1-EGFP electroporation compared to the small number of these cells in the pCAG-EGFP electroporated animals (***P<0.001; Student´s t test; n = 3 animals per condition). D-H) Tbr1 overexpression significantly reduced the complexity of layer II-IV neurons in terms of the number of basal dendrites (P<0.001; F), dendrite length (P<0.05; G) and the area occupied by the dendrites (P<0.05; H). The data in F-H are the mean ± S.E.M. (n = 15 neurons per condition, Student´s t test). Scale bar, 100 μm. I-K) To visualize the distribution of cells in the cortical layers, P7 sections were immunostained with antibodies against GFP and Cux1 (I-J). (K) As seen in the graph the percentages of GFP^+^Cux1^+^ cells were similar in control and Tbr1 overexpressing animals. (L) Almost 100% (98.8%) of the GFP^+^ cells were found in layers II-IV in the control animals whereas in the pCAG-Tbr1-EGFP electroporated animals, 82.5% and 13.3% of the GFP^+^ cells were located in layers II-IV and V-VI, respectively. However, these differences did not achieve statistical significance. Scale bar, 200 μm.

**Fig. 7** Tbr1 misexpression increases the proportion of Sox5 cells in the upper and deep layers of the neocortex. Coronal sections from P7 animals electroporated with the pCAG-EGFP or pCAG-Tbr1-EGFP plasmid were dual immunostained with specific antibodies against GFP and Sox5 (A´-B). Examples of GFP^+^Sox5^+^ double-labelled cells are indicated with white arrows (A´-A, B´-B and top insets) whereas an example of GFP^+^Sox5^-^ cell is indicated with yellow arrows (A`-A and lower insets). As there were very few GFP^+^ cells from pCAG-EGFP electroporated mice in layer VI these are not shown (see Fig. 6K). The cells marked by arrows in layers II-III are shown at a higher magnification in the insets. The pictures were composed of several images stitched together using the ImageJ software. Tbr1 overexpression increased the percentage of Sox5^+^ cells in layers II-III (70%, *P<0.05 Student´s t test), layers IV-V (77%, **P<0.05 Student´s t test), and in layer VI (37%, P = 0.05). The data are the mean ± S.E.M (n=3 animals per condition). Scale bar (shown in A´), 50 μm; 34.8 μm (insets).
