# Supplementary figures and images for: Tbr1 Misexpression Alters Neuronal Development in the Cerebral Cortex
Source: Mol Neurobiol. 2022 Jul 4;59(9):5750–65. doi: 10.1007/s12035-022-02936-x (PMC9395452; doi:10.1007/s12035-022-02936-x)

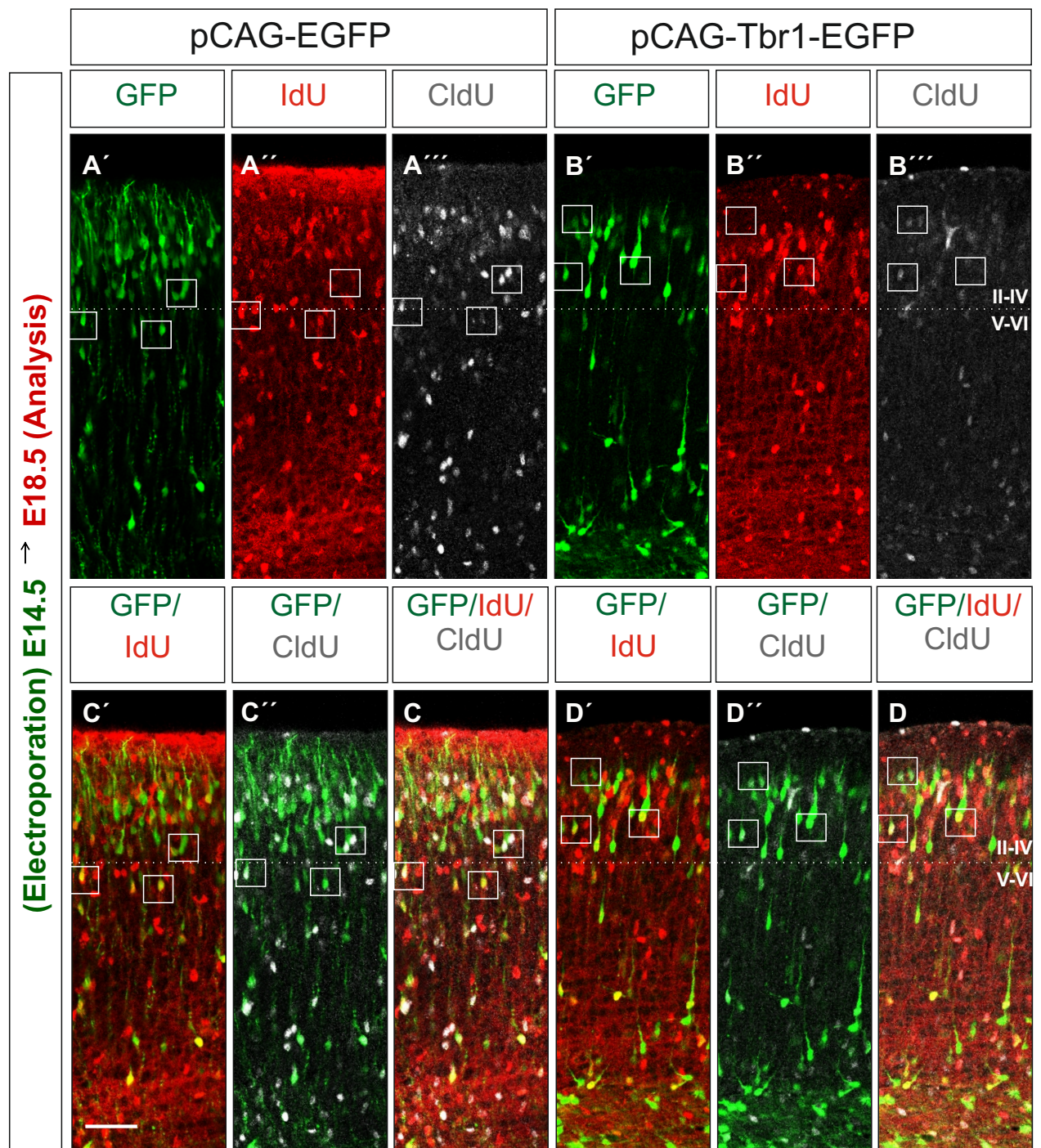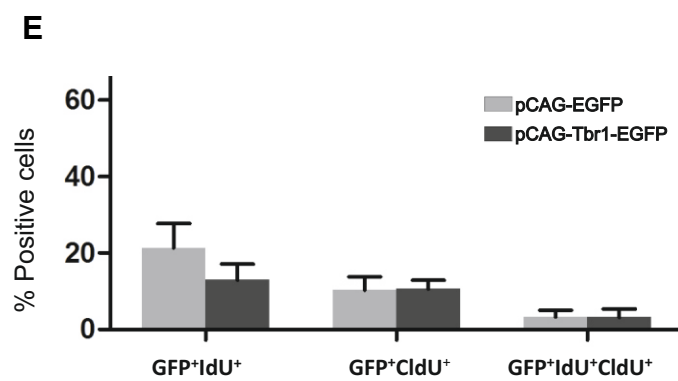

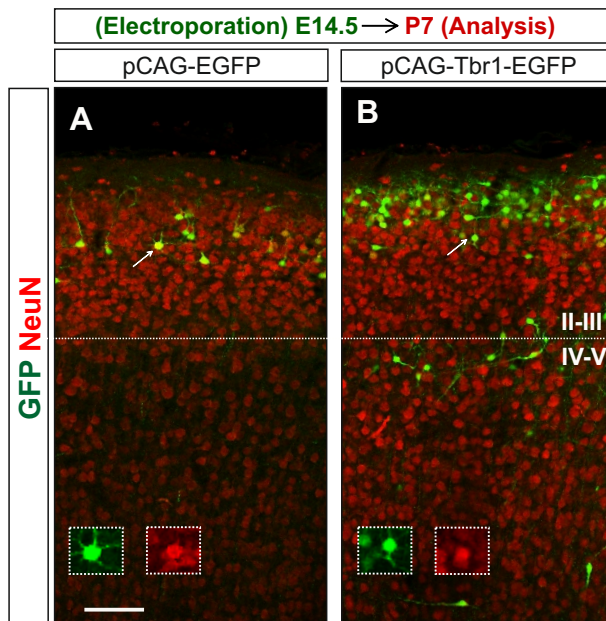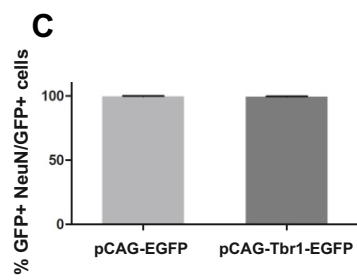

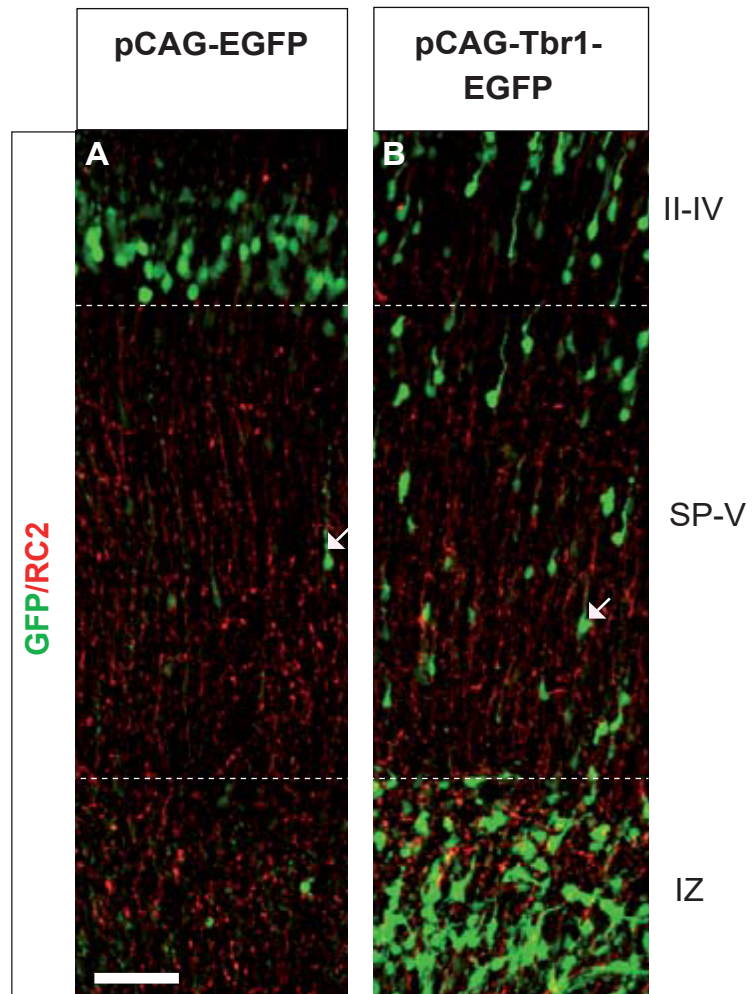

(Electroporation) E14.5 → P7 (Analysis)

pCAG-EGFP

pCAG-Tbr1-EGFP

A

B

GFP Tbr1

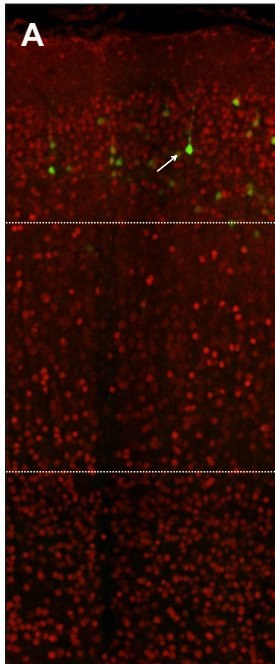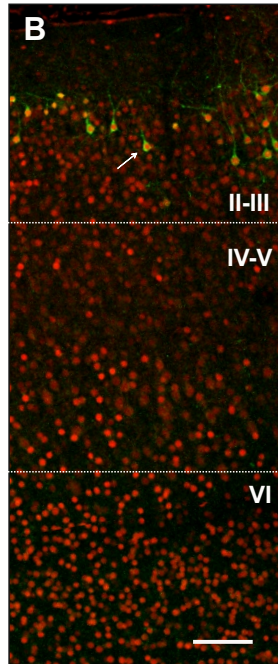

Supplement: Supplementary file 2 — Supplementary file2 (PDF 6494 KB) [file 12035_2022_2936_MOESM2_ESM.pdf]
